# Multisensory integration in the mammalian brain: diversity and flexibility in health and disease

**DOI:** 10.1098/rstb.2022.0338

**Published:** 2023-09-25

**Authors:** Ilsong Choi, Ilayda Demir, Seungmi Oh, Seung-Hee Lee

**Affiliations:** ^1^ Center for Synaptic Brain Dysfunctions, Institute for Basic Science (IBS), Daejeon 34141, Republic of Korea; ^2^ Department of biological sciences, KAIST, Daejeon 34141, Republic of Korea

**Keywords:** multisensory integration, sensory processing, flexibility, autism spectrum disorder, schizophrenia

## Abstract

Multisensory integration (MSI) occurs in a variety of brain areas, spanning cortical and subcortical regions. In traditional studies on sensory processing, the sensory cortices have been considered for processing sensory information in a modality-specific manner. The sensory cortices, however, send the information to other cortical and subcortical areas, including the higher association cortices and the other sensory cortices, where the multiple modality inputs converge and integrate to generate a meaningful percept. This integration process is neither simple nor fixed because these brain areas interact with each other via complicated circuits, which can be modulated by numerous internal and external conditions. As a result, dynamic MSI makes multisensory decisions flexible and adaptive in behaving animals. Impairments in MSI occur in many psychiatric disorders, which may result in an altered perception of the multisensory stimuli and an abnormal reaction to them. This review discusses the diversity and flexibility of MSI in mammals, including humans, primates and rodents, as well as the brain areas involved. It further explains how such flexibility influences perceptual experiences in behaving animals in both health and disease.

This article is part of the theme issue ‘Decision and control processes in multisensory perception’.

## 1. Introduction

In the mammalian brain, cortical areas interact with each other and communicate with subcortical areas through mutual projections. These complex circuits process a variety of sensory inputs and meaningfully integrate them to animals better understand complicated sensory information. How multisensory inputs are integrated in the brain, a.k.a. multisensory integration (MSI), is critical for an animal to make optimal and flexible decisions in a sensory world that is constantly changing. So far, many brain areas have been identified as polymodal areas that respond to a variety of different modality inputs. MSI, however, is beyond simple responses to multiple stimuli. It explains why neuronal responses to spatially and temporally co-presented multisensory stimuli differ from those to unisensory stimuli. MSI shapes not only neural responses but also the perceptual experiences of an animal by integrating one modality with another in stimuli given together. Which parts of the brain are engaged in this processing? In this review article, we investigated the important brain areas implicated with MSI (table 1). We particularly focused on audiovisual MSI (integration of auditory and visual stimuli) since both types of stimuli are physically discrete and separable when presented to animals at different times and in different spaces.

**Table 1.** Summary table of the references listed in this paper. References were classified according to the examined brain areas and the multisensory integration (MSI) types in health and diseases. ASD, autism spectrum disorder; SC, superior colliculus; PPC, posterior parietal cortex; STS, superior temporal sulcus; PFC, prefrontal cortex; MPC, medial prefrontal cortex; IFG, inferior frontal gyrus; ACC, anterior cingulate cortex; V1/VC, (primary) visual cortex; V1/AC, (primary) auditory cortex.

factor	brain									
		association cortex					sensory cortex		subcortical	
	SC	PPC	STS	insula cortex	PFC (MPC, IFG)	ACC	V1/VC	A1/AC	thalamus	amygdala
simple MSI	[1–18]	[19–21]	[22,23]				[24–29]	[26,28,30–33]	[34–38]	[39–41]
temporal MSI			[23,42]				[42,43]	[32,42,44,45]		
spatial MSI		[46]	[47,48]					[49]	[35]	
object MSI		[50–52]	[51,53–58]		[51,52,56,59,60,61]	[59,61]				
speech MSI	[62]		[55,62–67]		[68–71]		[62]			
ASD	[72]		[73]	[74]			[73]	[73]		
schizophrenia			[75]		[75]		[76]			

**Figure 1.**
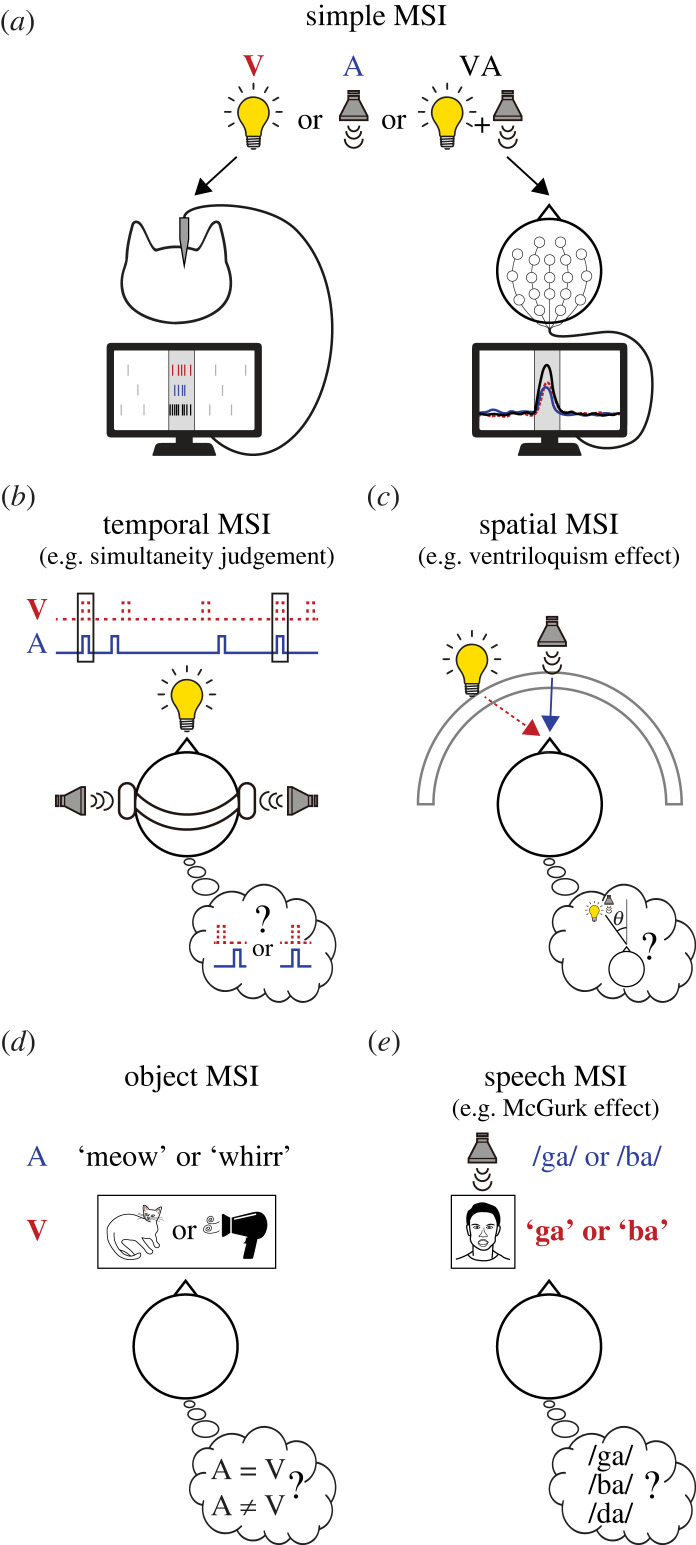
Task paradigms used for studying different types of multisensory integration (MSI). (*a*) Schematic illustration of simple MSI measured in subjects (either humans or animals) presented with random audiovisual stimuli simultaneously. (*b*) Temporal MSI as shown in the simultaneity judgement task. Red bold text and dashed lines, visual; blue regular weight text and continuous lines, auditory. (*c*) Spatial MSI as shown in the ventriloquism effect. (*d*) Object MSI by using congruent or incongruent multisensory stimuli. (*e*) Schematic illustration of speech MSI as shown in the McGurk effect.

MSI may happen in various circumstances where animals have different knowledge of the stimuli from previous experiences. Therefore, it is important to understand the types of MSI we are discussing. We classified MSI into five separate categories based on the parameters of the stimuli given to the animal (figure 1). First, many studies recorded brain activities or neural responses to simple multisensory stimuli in order to investigate the differences in responses evoked by multisensory stimuli compared to unisensory stimuli. We note these as simple MSI (figure 1*a*). Second, MSI can occur in the temporal dimension, which we note as temporal MSI (figure 1*b*). It requires the integration of audiovisual stimuli that are given at the same or different times. Third, we discuss spatial MSI, which requires the integration of audiovisual stimuli at different locations in space (figure 1*c*). These three types of MSIs happen even when the stimuli are novel to the animals. On the other hand, animals may integrate meanings in the stimuli that they learned from previous experiences. We examine two types of semantic MSI: those linked to the meanings of objects (object MSI; figure 1*d*) and those related to speech (speech MSI; figure 1*e*).

MSI varies subjectively, as individuals report different experiences even from the same multisensory stimuli. In this paper, we describe not only five types of MSI and the brain areas involved but also how MSI may change flexibly based on the states of an animal (mostly human cases). In §3, we discuss how MSI alters flexibly, proposing potential factors that affect MSI measured both behaviourally and physiologically. To gain more insights into the importance of such flexible MSI in normal brain functions, we extend our discussion on how neuropsychiatric disorders, such as autism spectrum disorder (ASD) and schizophrenia, influence MSI (see §4). We explain how each disease affects MSI in brain areas such as the sensory cortex, superior colliculus (SC), superior temporal sulcus (STS), insula cortex and prefrontal cortex (PFC), which results in abnormal MSI behaviours (table 2). In each section, we attempt to describe human studies together with relevant animal research.

**Table 2.** Abnormal MSI in different types of audiovisual integration tasks shown in patients with autism spectrum disorder (ASD) or schizophrenia.

task type	disease type	autism spectrum disorder (ASD)	schizophrenia
temporal MSI	simultaneity judgement task	temporal window ↑ ASD [77,78]; schizophrenia [79,80]	
	sound-induced flash illusion task	flash-beep illusion ↑ [81]	flash-beep illusion ↓ [82]
	temporal order judgement task	temporal window ↑ sensitivity ↓ [83,84]	sound → visual temporal sensitivity ↑ [85]
spatial MSI	spatial ventriloquist task	n.a.	no difference [86]
object MSI	object integration task	no difference [87]	n.a.
speech MSI	McGurk effect task	McGurk effect ↓ ASD [88–91]; schizophrenia [92–94]	

## 2. Brain regions involved in multisensory integration

**Figure 2.**
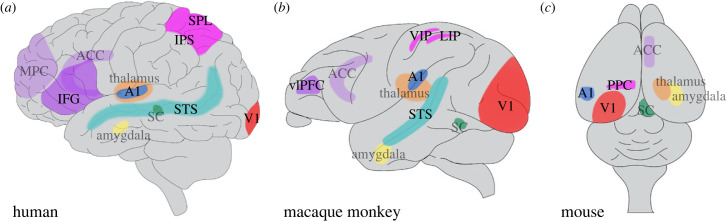
Multisensory brain areas. (*a*–*c*) Schematic overview of brain areas that process multisensory information. The equivalent cortical and subcortical multisensory areas were labelled in human (*a*), macaque monkey (*b*) and mouse (*c*) brains. These areas were selected based on data from electrophysiological recordings, functional imaging and anatomical tracing studies in these animals. Purple, frontal areas; MPC, medial prefrontal cortex; IFG, inferior frontal gyrus; ACC, anterior cingulate cortex; vlPFC, ventrolateral prefrontal cortex. Magenta, parietal areas; PPC, posterior parietal cortex; SPL, superior parietal lobule; IPS, intraparietal sulcus; VIP, ventral intraparietal area; LIP, lateral intraparietal area. Cyan, temporal area; STS, superior temporal sulcus. Red, primary visual cortex (V1); blue, primary auditory cortex (A1). Subcortical structures: orange, thalamus; green, superior colliculus (SC); yellow, amygdala. Areas residing inside the cortical surface (thalamus, amygdala, SC, MPC and ACC) are coloured transparently. (Online version in colour.)

On the way to deciphering the neural basis of MSI, anatomical tracing, electrophysiology and neuroimaging experiments revealed several key areas in the mammalian brain (figure 2). The first brain area where MSI was identified is the SC. Early studies in cats and primates identified the role of the SC in the accurate orientation of the multisensory stimuli in space. Not only the SC but several cortical areas were also identified as hotspots for MSI. One is the association cortex, which receives convergent inputs from multiple sensory cortices. These association cortical areas are scattered across the parietal, temporal and frontal lobes. For example, the parietal areas, the ventrolateral prefrontal cortex (vlPFC) and the STS receive visual and auditory inputs directly [95–97]. Furthermore, the primary sensory cortices have been shown to respond to other modality information through mutual interactions [30,98]. Beyond the cortex, even sensory thalamic nuclei were involved in processing multisensory information [34]. Therefore, MSI occurs in multiple brain areas in parallel, and this might be important for the flexible integration of sensory stimuli that constantly bombard an animal with distinct features in space and time. In this section, we tried to cover not all but most of the important brain areas that are known to be involved in MSI.

### (a) Superior colliculus

The first brain area systemically examined as a potential hub for MSI is the SC [99]. The SC is a subcortical midbrain structure largely known for its importance in gaze orientation towards visual stimuli [100]. The pioneering work to identify multisensory neurons in the brain was performed in the SC of anaesthetized cats using single-unit recordings [1], and similar multisensory neurons were found later in the SC of rhesus monkeys [2,3] and alert cats [4] (figure 1*a*). While neurons in the superficial layers of the SC were primarily involved in visual responses, multisensory neurons were in the deep layers of the SC [1,3,5]. These neurons changed their responses (either enhanced or depressed) to the multisensory stimuli compared with their unisensory responses depending on the configuration of the stimuli (e.g. stimulus combination, timing or physical properties). One important MSI property of the SC neurons was nonlinear multisensory responses, following the principle of inverse effectiveness, which explains that the multisensory responses of the SC neurons were more robust when the unisensory stimuli that evoked less response were combined [1,4,6,7]. Another interesting observation was that the multisensory activities of deep-layer SC neurons were highly dependent on spatial and temporal coincidence (see §3c below) [3,4,7,8]. The inverse effectiveness and the spatial matching principles of MSI in the SC were also demonstrated in mice through large-scale electrophysiology and modelling of a neural population with nonlinear audiovisual responses [9].

SC receives auditory and visual inputs from a variety of cortical (both sensory and association) and subcortical (such as the brachium of the inferior colliculus) areas [101], and these converging inputs may be critical for shaping the multisensory responses of the SC neurons. For example, two cortical areas projecting onto the SC, the anterior ectosylvian sulcus (AES) [102,103] and the rostral lateral suprasylvian sulcus (rLS) [104,105], were found to influence the multisensory properties of the cat SC. A reversible deactivation of these areas disturbed multisensory orientation behaviours in cats [106,107] and abolished the multisensory activities in the SC [10–12]. Furthermore, the multisensory activities of the SC did not develop if these cortical areas were removed in the early development of cats [13]. The SC is an output area that projects directly to the motor and premotor neurons in the brainstem or the spinal cord, and a majority of these descending projections were more active in response to the multisensory stimuli [5]. The SC multisensory neurons were critical for the multisensory behaviours, as lesions of the SC in cats demolished enhancement and depression of orientation behaviours by multimodal stimuli, although the modality-specific orientation behaviours were maintained [108]. Therefore, the multisensory activity of the SC neurons is potentially important for accurate orientation responses of animals in the context of real-life multisensory environments.

### (b) Parietal cortex

Areas of the parietal cortex have been found to be involved in MSI. Diffusion tensor imaging has shown that individuals with higher white matter connectivity of the superior parietal lobule (SPL) and the intraparietal sulcus (IPS) with early sensory areas displayed faster detection of the multisensory stimuli [109]. Furthermore, intracranial recordings in human SPL found nonlinear multisensory interactions between simple auditory and visual stimuli [19]. Through fMRI in humans, the SPL and the IPS were also found to be involved in integrating multisensory motion stimuli, their responses being stronger to congruent audiovisual motion [110]. Moreover, the IPS was shown to be involved in both visual and auditory motion discrimination, and its activity was enhanced during crossmodal comparison of motion speeds [111]. MSI in these areas was not straightforward but further modulated by stimulus or behavioural conditions. For example, Regenbogen *et al*. have demonstrated that the IPS is more involved in processing noise-rich audiovisual stimuli, while noise-free stimuli were integrated at early sensory cortices, suggesting a specific role of the IPS in more difficult MSI [50]. Other studies have shown that the IPS weighted sensory signals according to the sensory reliability and the task relevance in a ventriloquism-like spatial localization task [46] and that super/sub-additivity of the IPS response to multisensory stimuli predicted the accuracy of subjects' performance in a semantic object categorization task [51].

The intraparietal cortices, such as the ventral intraparietal area (VIP) and the lateral intraparietal area (LIP), were also shown to be candidate areas for MSI in primates. The VIP was found to display visual–tactile MSI [112–113]. Spatial and temporal congruency between visual and tactile stimuli was important for the MSI effects on the VIP responses, which followed the principle of inverse effectiveness [112]. Not only visual–tactile but auditory responses were observed in the VIP. The receptive fields of visual and auditory stimuli were found to be highly overlapping in space across the VIP neurons, and the VIP neurons represent modality-invariant external space [114]. Furthermore, similar areas within the borders of VIP were found to be activated in response to both visual and auditory moving stimuli, although the visual activation was much more robust [115]. Visual and auditory responses were also found in the LIP. In monkeys performing memory-guided saccades to unimodal visual or auditory cues, spatially tuned bimodal neurons in the LIP showed activity in both sensory-guided tasks [116,117]. However, so far, studies have just found that neurons in the VIP and the LIP show their bimodal responses to visual and auditory stimuli, not their roles in audiovisual MSI or any changes in their responses to audiovisual multisensory stimuli. Future studies are required to understand the audiovisual MSI in these areas more clearly.

Due to its connectivity with sensory, motor and association areas, the posterior parietal cortex (PPC) is also regarded as a potential MSI region in rodents [118,119]. Song *et al*. found that feedforward inhibition in the mouse PPC is responsible for auditory dominance in go/no-go decisions under audiovisual conflicts [20]. However, in rats performing categorization-based left–right decisions, Raposo *et al*. showed that inactivation of the PPC did not affect the multisensory decisions enhanced by congruent audiovisual stimuli but only impaired the visually guided performance [120]. In another study, mice made left–right choices when they detected changes in visual or auditory stimuli. Inactivation of the PPC did not affect their behavioural choices in response to changes in either auditory, visual or audiovisual stimuli [121]. Therefore, the rodent PPC may primarily process visual information for making decisions. This process can be inhibited by incongruent auditory stimuli presented together with visual stimuli that lead to go/no-go action decisions [20,21].

### (c) Temporal cortex

Another well-known cortical area that displays MSI is the temporal cortex [122]. Within this region, the STS has been recognized as a critical area involved in MSI. *In vivo* single-unit recordings in macaque monkeys revealed multisensory neurons in the STS [22,23]. For example, the STS neurons that showed visual responses were further modulated by sound stimuli congruent with the visual stimulus [22]. In humans, fMRI and PET imaging also revealed activation of the STS during semantic integration of audiovisual stimuli [63–65]. In the year 2000, Calvert and colleagues reported fMRI experiments with semantically matching and non-matching audiovisual stimuli. They found that the matching audiovisual stimuli evoked supra-additive enhancement, and the non-matching stimuli evoked sub-additive suppression of the activity in the left STS [63]. Moreover, other lines of studies showed that higher activity in the left posterior STS was responsible for a stronger McGurk illusion, an illusory speech perception induced by a mismatch between the sound and the lip shape saying a syllable, suggesting a role of the STS in generating the McGurk illusion of the conflicting audiovisual stimuli [64,65] (figure 1*e*). Supporting this, disruption of the STS by fMRI-guided transcranial magnetic stimulation caused a reduction of the McGurk effect [66].

Although many human brain imaging studies have examined audiovisual integration in the STS for speech perception (figure 1*e*), some studies found that the STS is also involved in MSI for perceiving non-speech objects [53,54] (figure 1*d*). In fMRI experiments by Beauchamp and colleagues, subjects were presented with an animal image, a man-made object or meaningless scrambled visual stimuli. In some trials, the visual stimuli were presented together with sound stimuli related to the visual objects or meaningless ripple sounds, either congruently or incongruently. Interestingly, posterior STS showed higher responses to meaningful unimodal stimuli and congruent audiovisual stimuli compared to meaningless ones and incongruent combinations [53]. Furthermore, Berger and colleagues found through an fMRI study that the STS was also active during the integration of an imaginary visual object and real auditory stimulus in a space, which evoked a ventriloquist illusion—an illusory sound localization to the place where the visual cue was present [47] (figure 1*c*). Like other multisensory areas previously described, multisensory response in the STS also showed the inverse effectiveness in both the speech and the object audiovisual integration conditions [55].

### (d) Frontal cortex

The areas in the frontal lobe, particularly the premotor and prefrontal cortices, have been suggested as potential areas for MSI due to their rich connections with other cortical and subcortical areas. For example, the frontal cortex was found to be connected with STS and the superior temporal gyrus (STG), which are well-known multisensory areas [123,124]. By making single-unit recordings in macaque monkeys receiving unimodal and multimodal face/non-face visual and corresponding vocalization/non-vocalization auditory stimuli, Sugihara and colleagues have shown that the neurons in the vlPFC respond not only to unimodal visual and auditory stimuli [125,126] but also to multimodal stimuli [68]. Multisensory neurons that showed both enhancement and suppression in their multisensory response were found in the vlPFC, although multisensory suppression was more prominent [68]. Unlike the parietal or temporal association areas, the vlPFC showed more selective responses to the face/vocalization multisensory stimuli rather than non-face/non-vocalization, demonstrating for the first time that single prefrontal neurons can integrate audiovisual communication information [68]. In a human fMRI study using linguistic visual and auditory stimuli consisting of words, phrases and sentences, the activity in the ventral region of the left inferior frontal gyrus (IFG) was important for the integration of audiovisual inputs during sentence comprehension [69]. In another human fMRI study focusing on the processing of the phonetic features of audiovisual speech using phonetically matching or conflicting vowel stimuli (with no McGurk effect), the inferior frontal region—specifically Broca's area—was found to increase activity in response to conflicting stimuli [70].

Another frontal cortex area that is potentially important for MSI is the anterior cingulate cortex (ACC). One human fMRI study used objects or scenes for the audiovisual stimuli and found that the ACC and the medial prefrontal cortex (MPC) showed strong multisensory responses to congruent audiovisual stimuli [59]. Another human fMRI study showed that the ACC was activated by both auditory and visual unimodal motion stimuli and displayed activity enhancement in an audiovisual speed comparison test, suggesting the role of ACC in MSI [111]. In mice, ACC showed a reciprocal connection with different sensory cortices [119]. Therefore, it is possible that the ACC is a potential candidate for MSI and processes and integrates sensory information across modalities. Future studies are required to understand the role of ACC in MSI.

### (e) Primary sensory cortices

Although many multisensory studies have focused on the higher association cortices, more recent studies have found that lower-level sensory areas, previously thought to be strictly unimodal, also contribute to MSI. Anatomical connectivity between the sensory cortices and other known multisensory areas has been observed [119,127–135]. For example, tracing experiments in non-human primates [129,130] and rodents [119,132,134] have shown that the primary visual cortex (V1) is connected to low-level auditory areas, including the primary auditory cortex (A1). In humans, connectivity between visual and auditory cortices was also demonstrated through diffusion tensor imaging [127]. However, the effect of cross-modal inputs in sensory cortices can be additive or suppressive depending on the recipient modality and the stimulus configurations. Here, we describe some examples reported so far.

*In vivo* neural recordings in rodents [24,44,98] and non-human primates [31,32] revealed that sensory inputs from other modalities modulated neuronal activity in the primary sensory cortices. Recordings in the A1 of awake Mongolian gerbils presented with combined flash and sound burst stimuli found enhancement or suppression of auditory responses in more than a quarter of the recorded neurons, and this modulation was more prominent when the two stimuli were temporally closer and when the visual stimulus is given first [44]. Similar results were found by Kayser and colleagues in the A1 of rhesus monkeys presented with naturalistic audiovisual stimuli [32], and they showed that this multisensory modulation of A1 neurons was dependent on the congruency of the audiovisual stimuli and followed the principle of inverse effectiveness [31,32]. Furthermore, Atilgan *et al*. found that temporally matching visual stimuli facilitated a more accurate representation of the auditory information in the A1 via direct inputs from the visual cortex (VC) [30].

Through various electrophysiological recordings in mice, Iurilli and colleagues showed that activation of the A1 inflicted GABAergic inhibition on the V1, and visual responses were decreased in response to audiovisual stimuli [24]. Moreover, they showed a reflection of this on behaviour, as mice showed reduced response to a conditioned visual stimulus when the visual stimulus was temporally coupled with an auditory stimulus [24]. Suppression of visual responses in V1 of mice by long-range projections from auditory cortex (AC) was also demonstrated by Garner and Keller, by coupling preceding unimodal audio cues to unimodal visual stimuli in a behaviourally relevant context [98]. This effect was found to be experience-dependent and occurred only after learning [98]. Furthermore, a two-photon calcium imaging of V1 neurons in awake mice showed that their responses were modulated (either enhanced or suppressed) by audiovisual stimuli depending on the configuration of the multisensory stimuli such as composition and contrast, as well as temporal congruency [25].

Audiovisual MSI in low-level sensory cortices has also been investigated through haemodynamic and electromagnetic brain imaging methods. In both V1 and A1, early cross-modal activations and audiovisual interactions were observed through MEG and fMRI in humans [26]. Phase-modulation of visual responses in the VC areas, including the V1, was enhanced by the addition of the same-phase auditory stimuli as measured in humans by EEG [43] and subdural recordings [27]. Similarly, auditory responses in the AC areas, including the A1, were found to be enhanced by visual stimuli through an fMRI study in macaque monkeys [33] as well as an EEG experiment in humans [45].

### (f) Thalamus

The sensory cortices are connected not only to other cortical areas but also to subcortical structures like the thalamus [132]. Sensory thalamic nuclei project not only to the cortical region of their own modality but also to the other primary sensory areas, showing a potential role of MSI [132,136]. Single-neuron recordings in rats performing an auditory spatial discrimination task with or without visual cues found that auditory thalamic neurons responded to not only auditory but also audiovisual stimuli and that the early phase of auditory thalamic responses was driven by visual signals [35]. Multisensory activity in the sensory thalamus was also observed in human subjects. In an fMRI study on human subjects judging whether two bars passed or collided with each other, a collision sound presented together with visual stimuli in some trials evoked more activity in the posterior thalamus when subjects perceived collision in audiovisual trials [36]. Another fMRI study by Noesselt *et al*. has shown that visual and auditory thalamic nuclei are involved in the enhanced detection of low-contrast visual stimuli by auditory co-stimulation [34]. Furthermore, van den Brink and colleagues found that the connectivity in the auditory stream from the cochlear nucleus to the auditory thalamus and to the A1, measured by diffusion tensor imaging, can predict a human's ability to combine visual and auditory information during an audiovisual integration task [37]. Collectively, these studies support the idea that sensory thalamic nuclei not only relay specific modality information to their corresponding cortices but are also directly involved in MSI processing.

Due to its connectivity to sensory, multisensory and premotor areas [137–140], the medial pulvinar nucleus of the thalamus has also been suggested as a potential MSI region (see more details in the review paper by Froesel *et al*. [141]). Auditory [142] and visual [143] responsive neurons were found in the pulvinar in separate studies. More recently, single-unit recordings in macaque monkeys have revealed multisensory neurons that show suppressive responses to multisensory stimuli [38]. In this study, they also identified ‘complex multisensory’ neurons, whose activity depended on the categories of the stimuli [38]. Therefore, the pulvinar might be one of the thalamic areas that play a critical role in MSI.

### (g) Amygdala

Animals must make appropriate reactions for survival in an environment with dangerous stimuli. The pathways processing threatening stimuli from each sensory modality have been shown to project onto the amygdala [144–146], a core midbrain structure that is important for emotional processing and fear responses [147]. Human studies have shown that lesions of the amygdala decrease the auditory recognition of fear and anger intonations [148] and the visual detection of angry, threatening faces [149]. Information conveying threats rarely comes from a single modality, making MSI in the amygdala potentially valuable for survival. Very recently, Shan *et al*. have investigated the active participation of the amygdala and its close regions in audiovisual MSI through multi-array recordings in macaque monkeys by presenting them with auditory, visual, and audiovisual looming and receding stimuli [39]. Both positive and negative modulation of sensory responses (mostly auditory responses) was observed in the amygdala neurons when the audiovisual stimuli were presented [39]. Human fMRI studies also showed that the amygdala is involved in the facilitation of fear perception in the face when the visual stimulus is paired with an emotionally congruent rather than an incongruent fearful auditory stimulus [40]. Furthermore, the amygdala showed stronger responses to audiovisual (face–voice) emotional stimuli compared with unimodal emotional stimuli, independent of the types of emotion [41]. Further research is required to understand the role of the amygdala in the MSI of emotional stimuli in more detail.

## 3. The flexibility of multisensory integration and decision makings

In a rapidly changing environment, one of the most important issues for animals would be adapting their behaviours according to given sensory inputs that vary in time and space across modalities. Integration of different modality inputs is important for animals to make optimal decisions in a multisensory environment. Therefore, the integration process needs to be flexible in a multisensory environment, according to the animal's state. So far, we have described brain areas spanning the cortical regions, from the higher association cortex to the lower sensory cortex and subcortical areas, including the colliculus and thalamus, and the unique or similar principles of MSI. Previous studies in humans, however, found huge variations in multisensory perception of the McGurk illusion and Colavita visual dominance during the detection of audiovisual stimuli across individuals and experimental conditions [150–153]. A human twin study revealed that shared genetic background was not sufficient to explain all individual differences in McGurk effects, as the authors observed wide variations of the McGurk effect even in twins [154]. These variations naturally suggest that MSI is not uniformly developed across individuals. Rather, it can be modified during development as well as by experience and learning throughout life [155]. The flexible change in MSI can happen even in a faster time window with state changes in individuals [156]. However, the neural mechanisms underlying the flexibility in MSI are still not fully understood.

**Figure 3.**
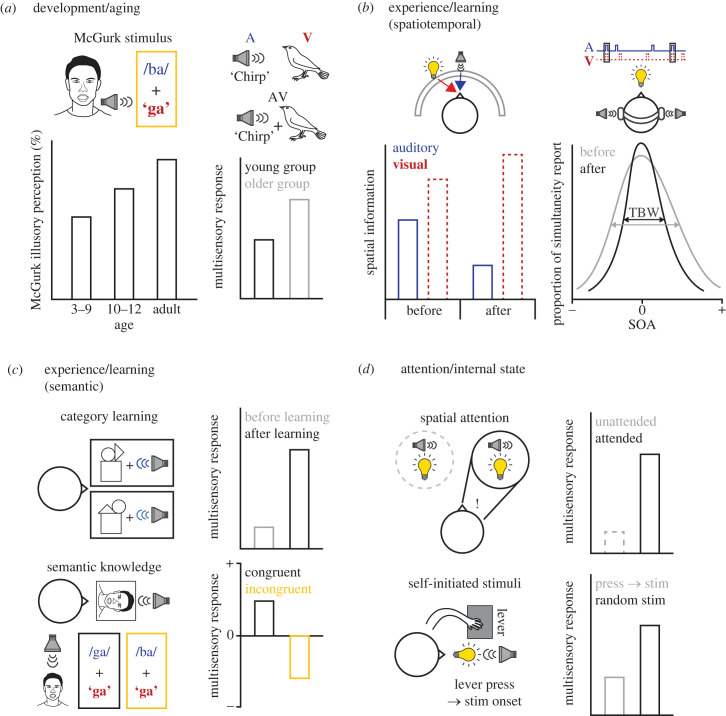
Factors that cause flexible changes in MSI. (*a*) Left, age-dependent change of McGurk illusory perception [157]. Note that there is a gradual increase in illusory perception with increasing age. Right, the multisensory response of young adults (black) and older adults (grey) to audiovisual stimuli [52]. (*b*) Spatio-temporal modulation of MSI by experience and learning during the task. Left, spatial information of each modality before and after experience or training [158–161]. Right, the proportion of simultaneity reports before and after the training at different stimulus-onset asynchronies (SOAs) [42,162,163]. Arrows indicate temporal binding window (TBW). Blue regular weight text and continuous lines, auditory stimuli; red bold text and dashed lines, visual stimuli. (*c*) Modulation of MSI by semantic experience and learning. Top, multisensory responses in the STS, IFG and IPS during audiovisual category learning [56]. Bottom, multisensory responses in the STS to a congruent (black) or an incongruent (yellow) speech [63]. (*d*) Top, effects of the top-down spatial attention on multisensory responses in the brain [164–167]. Grey, multisensory response to an unattended stimulus; black, multisensory response to an attended stimulus. Bottom, multisensory responses to expected multisensory stimuli after lever press (grey) or unexpected onset of stimuli (black) [168]. stim, stimulus.

This section introduces the three categories that can induce flexible changes in MSI: development/ageing (figure 3*a*), experience/learning (figure 3*b*,*c*) and attention/internal states (figure 3*d*). We have mainly described the flexibility of MSI found in humans, with an emphasis on the temporal, spatial and semantic (both object and speech) MSI (figure 1). We have also introduced animal studies, which pioneered a path for understanding the neural circuit mechanisms of flexible MSI by using state-of-the-art methods.

### (a) Development/ageing

As multisensory organisms, animals already develop their multisensory processing ability in the prenatal stage [169]. Along with the gradual development of unisensory systems [14,170,171], multisensory systems also develop postnatally [14,99]. Pioneering studies on the development of multisensory processing found age-dependent changes in MSI in cat brains [14,99]. For example, Wallace and colleagues conducted electrophysiological recordings in the cat SC throughout postnatal development. There was a gradual increase in the number of multisensory neurons, and the multisensory responses in the SC neurons increased as cats became older [14]. A similar result was also found in the SC of the monkey [15]. Furthermore, early visual deprivation in cats by raising them in a dark room when they were young caused a significant decrease in multisensory responses in the SC and AES [16,172]. The AC of human patients with congenital cataracts at an early age also showed a reduced multisensory response, while abnormal visual suppression by co-presented sound was observed in the VC [28]. Patients suffering cataracts at an early age also showed deficits in MSI, as their visual perception was less affected by a distractor tone than in normal people [173]. These studies provided direct evidence of the gradual emergence of multisensory neurons in the brain and the importance of sensory experiences during postnatal development.

Several studies measured the behavioural reactions of human infants to multisensory stimuli and established a canonical perspective on how the ability of MSI develops in the brain [155]. Multisensory perceptual narrowing theory explains the development of MSI as occurring in three stages: immature, broadly tuned and narrowly tuned stages [155]. During the immature stage, non-verbal infants (less than one month old) have preferential reactions to multisensory stimuli [174,175]. In this stage, infants preferentially respond to multisensory stimuli based on intensity-matching or temporal synchrony [175,176], although the dependency of their multisensory preference on the spatio-temporal synchrony of multisensory stimuli was relatively low in this stage [174,177–179]. Moreover, human infants in the immature stage tend to react equally to unnatural multisensory stimuli (e.g. monkey faces with arbitrary tones) [174]. These results indicate that MSI is an innate feature of the brain and non-specifically happens in the early developmental period regardless of spatio-temporal asynchrony between modalities. With exposure to stochastically frequent multisensory stimuli that are spatio-temporally coherent and possess natural meanings between modalities, human infants develop some degree of specificity in MSI. As a result, human infants in the broadly tuned stage (five to eight months old) showed a narrower temporal binding window [177,178] as well as increased attention to naturally coherent multisensory stimuli [180,181]. Moreover, infants at this stage develop an audiovisual perceptual ability to be more likely to respond to coherent multisensory stimuli and experience the McGurk effect [182]. In the narrowly tuned stage (approx. 12 months old), babies develop more narrowed MSI, preferentially perceiving and responding to coherent native speech [183,184] (figure 3*a*, left). Overall, human infants show flexible changes in MSI during postnatal development, such as perceptual narrowing in MSI by binding more specific and coherent multisensory stimuli. Further studies are required to help our understanding of the neural mechanisms underpinning such development.

Humans continue to show changes in MSI as they get older and become adults. For example, Robinson and Sloutsky showed age-dependent changes in the dominant modality for decision, as the children in their study (who were approx. 4 years old) were much more responsive to the auditory stimuli and usually showed auditory-based prediction for upcoming events [185]. Similarly, the McGurk effect and Colavita visual dominance effect were less prominent in the children, as visual information was less effective in manipulating their auditory perception [157,186]. These results indicate that humans develop more visually dominant MSI as they age. Even in simple MSI conditions, humans show more enhancement in their responses to multisensory stimuli than to unisensory stimuli with ageing. This improvement in MSI effects occurs even during the adolescent period, as human subjects showed faster reactions to multisensory stimuli than to unisensory stimuli as they aged during adolescence, and reached their maximum performance at the age of 14 [187]. These reports suggest that developmental maturation of the brain during adolescence is critical for the maturation of MSI.

There are also differences in MSI in fully grown adults according to age. For example, the PPC and the MPC of older adults responded more prominently to multisensory stimuli than to unisensory stimuli, whereas sensory areas showed weaker responses than in the younger group [52] (figure 3*a*, right). Moreover, only the older adults showed a significant correlation between the level of PPC and MPC activities and the ability to perceive multisensory stimuli [52]. These results suggest that the brain develops an adapted strategy with ageing to maximize the benefits of MSI [52,188]. However, older participants with minor hearing loss tended to show less auditory perception and showed smaller MSI for the spatial localization of the audiovisual stimuli [189] (figure 1*c*) while increasing the weight of visual modality for MSI [52]. Therefore, changes in MSI with ageing may result from changes in unisensory systems. Regardless, developmental maturation and ageing are critical factors for long-term adaptation and lifespan flexibility of MSI processing in the brain.

### (b) Experience/learning

Animals face numerous multisensory events from the environment throughout their life. From the perspective of the brain as a Bayesian receiver, the brain undergoes dynamic perceptual recalibration of the external world and internal priors according to experience [190–192]. A causal inference explained audiovisual spatial integration in humans by using maximum-likelihood estimation (MLE) about unisensory perception and an individual's prior beliefs [193,194] (but see [195] for inconsistency about the MLE). Therefore, prior experience would be one of the major factors that can cause flexible MSI. This section discusses how experience and flexible learning shape MSI in the brain. We divided the cases into three categories: non-instructive exposure to the stimuli without any feedback or rewards, instructive exposure with feedback during learning and effect of prior knowledge.

Repetitive exposure to multisensory events without any associated rewards can shape spatial MSI responses in humans [155]. For example, repetitive exposure to ventriloquism-inducing audiovisual stimuli without any feedback induced humans to perceive more illusory localization of the sound stimuli to the visual cues, which is called the ventriloquism aftereffect [158,159,196] (figure 3*b*, left). Hong and colleagues also found that repetitive exposure to spatio-temporally congruent or incongruent stimuli enhanced or suppressed the common cause prior (an assumption that different modality stimuli are from the same object) of human participants during spatial MSI [159]. Furthermore, multisensory exposure attenuated auditory sensitivity during spatial integration in the STG [48]. These studies indicate that multisensory exposures enhance the weighting of visual information during spatial MSI.

Other studies showed that the association of random auditory cues with visual objects disrupts visual processing in the brain [17,57,58,197,198]. For example, meaningless sound stimuli co-presented with visual objects induced robust multisensory responses in the human middle temporal gyrus (MTG) and STG [57,58]. Interestingly, the robustness of multisensory responses in the MTG is negatively correlated with the performance of subjects in the following visual discrimination and object categorization after the multisensory exposure, suggesting that multisensory exposure dampens visual recognition [198]. In other studies, repetitive audiovisual exposure for visual-to-auditory substitution switched visual-preferring activity to auditory-preferring activity in the MTG [197]. Changes in MSI in the brain were observed not only humans but also in other animals. For example, experiencing spatio-temporally overlapping multisensory stimuli but not the non-overlapping stimuli increased multisensory responses in the cat SC [17]. Recent rodent studies have also showed that repeated multisensory exposure drives a multisensory response in the VC [29]. However, it is still obscure how other higher brain areas are affected by multisensory exposure in animals.

Animals learn the meaning of the stimuli rapidly, not only as a result of simple exposure to the stimuli, but also after experiencing stimuli associated with specific outcomes makes. Such learning induces changes in MSI as the values of specific sensory information change in distinct sensory modalities. For example, when human and animal subjects learned to associate specific stimuli (either auditory or visual) with designated reward values, they recalibrated the weights of the stimuli according to the values during spatial integration in the ventriloquism task [160,161] (figure 3*b*, left). Similarly, learning the simultaneity judgement task (figure 1*b*) increased correct judgements of the timing in visual and auditory stimuli and sharpened the temporal binding window, during which the subject reported that visual and auditory stimuli are presented at the same time and integrated as a multisensory stimulus [42,162,163] (figure 3*b*, right). The enhancement of functional connectivity accompanied the sharpening of the temporal binding window among the STS, VC and AC [42]. Interestingly, such sharpening was not observed if the subject was not informed about the correctness of their judgements [42,162]. These results strongly suggest that it was not the simple exposure but the learning of the task that induced changes in MSI.

Semantic learning also changed MSI. One study in humans tested an audiovisual object association task, in which subjects needed to report whether or not the presented audiovisual stimuli were congruent while their brain activity was measured [56] (figure 1*d*). The brain develops multisensory responses in the STS, IFG and IPS during learning of the audiovisual category [56] (figure 3*c*, top). In the case of animal studies, the modulation of primary sensory areas by multisensory learning has been examined in rodents. Interestingly, when mice were trained to discriminate paired audiovisual stimuli, perceptual learning enhanced multisensory decisions and responses in both the AC [199,200] and the VC [98]. More animal studies are required to understand how learning changes MSI properties in the association cortex.

Lastly, another considerable aspect of the flexibility of MSI is that our brain processes the multisensory information depending on our prior knowledge. For example, it has been shown that working memory of a previous multisensory experience influences the multisensory responses of PFC neurons [60]. Multiple studies have conducted experiments on how the congruency between auditory and visual stimuli affects human multisensory perception (figure 1*d*,*e*). Interestingly, congruent and incongruent stimuli induced different responses in certain brain areas, and they are processed in different brain areas, including the STS and frontal areas [56,201]. A previous study showed that temporally and contextually congruent audiovisual speech enhanced cortical representation of speech [71]. For example, during speech perception, the STS area shows multisensory enhancement in response to congruent speech stimuli while exhibiting depressed multisensory responses to incongruent stimuli [63] (figure 3*c*, bottom). Moreover, in a series of studies, it has been established that congruent stimuli are mainly processed in the PFC and the STS, while incongruent stimuli induce strong responses in the ACC [56,61,63]. These data support the idea that the PFC and the ACC in the frontal area have roles in the semantic categorization of congruent and incongruent multisensory stimuli [56,61]. Meanwhile, the PPC processed both the congruent and incongruent stimuli equally [61]. Because the congruency between modalities is based on the subject's knowledge, it is highly plausible that MSI can flexibly change in the brain according to prior knowledge.

### (c) Attention/internal brain state

MSI is also affected by subjects' internal states. In the case of the McGurk illusory perception, some studies revealed that the level of illusory perception is highly correlated with pre-stimulus activity of the STS in humans [67,152]. The pre-stimulus activity of the STS affected the functional connectivity between the STS and the auditory areas, which influenced how much auditory information contributes to multisensory perception [67]. In general, pre-stimulus activity is highly associated with ongoing changes in internal states, which reflect attention, expectation, arousal and self-generated movements. Therefore, any factors that can change the internal states can strongly affect and modulate MSI.

Attention is a fundamental cognitive state of the brain formed by complex neural mechanisms, which select sensory elements for further processing among multiple elements presented [202]. From the beginning of the study of sensation and perception, attention has been considered one of the most decisive factors that control sensory processing. Attention can either be driven by external stimuli through bottom-up modulation or consciously directed in a top-down fashion [164]. For example, salient multisensory stimuli drove the bottom-up attention of the observer and improved the perception of individuals in [203,204]. Interestingly, spatio-temporally coherent multisensory stimuli attracted stronger bottom-up attention accompanied by saccadic movement and induced stronger responses of SC neurons to the stimuli than the incoherent stimuli [8,18,205,206]. Therefore, the attention recruited by stimuli in a bottom-up fashion can modulate MSI, and it is a mechanism that can selectively integrate important and relevant stimuli for the survival of an animal in a complicated environment with numerous sensory inputs.

It is well known that spatial attention reshapes the receptive-field properties in the lower sensory areas, including the VC and AC [62,207–209], which may enhance MSI in higher cortical areas. Supporting this idea, a series of studies have found how the instructed top-down spatial attention modulates MSI [164–166,210]. If multimodal stimuli were presented at the spatially attended area, the multimodal brain areas, such as the SC and the STS, showed robust integrative responses (figure 3*d*, top). Attention guided to specific sensory modalities can also affect MSI. For example, effective MSI was only observed when participants were guided to attend to both modalities [167,211,212]. Moreover, the level of attention to the multisensory stimuli showed a significant correlation with the enhancement in the perception of the multisensory information [211,213]. Attention to the given multisensory stimuli can be degraded if subjects are guided to attend to other tasks irrelevant to MSI [213,214]. For instance, human subjects experienced less sound-induced illusory flash perception (SIFI) if they were instructed to attend to other tasks, such as remembering letters given before the test stimuli [213]. In another study, both congruent and incongruent audiovisual stimuli accelerated reaction times if participants were instructed to report the onset of any stimuli (multisensory detection task) [214]. However, the incongruent stimuli caused an even slower reaction time than unisensory stimuli during the discrimination task, during which participants should report only the correct stimuli [214]. These studies show that MSI is highly influenced by the level of attention given to the stimuli or task paradigms.

On the other hand, one modality can be more potent than the other depending on which modality information is more attended to within the multisensory stimuli. For example, several studies have examined how the attention given to specific features of the stimuli, such as temporal information or spatial information, determines prepotent modality during MSI. When participants were asked to focus on temporal information, they showed more auditory dominance in perceiving audiovisual stimuli [165,215]. Conversely, attention to spatial information caused visually dominant perception in participants through modulation of neural response in contralateral AC and STG from the direction of biased perception [48,49,165,216]. These results support the ‘modality appropriateness’ theory, which explains how an animal uses modality-specific information to appropriately process specific features in the multimodal sensory information [217]. These results also show how top-down attention flexibly changes MSI in the brain.

In the spatial localization task (figure 1*c*), human subjects showed less audiovisual integration if the stimuli were generated by themselves [168] (figure 3*d*, bottom). Therefore, multisensory perception may be affected by a corollary discharge signal in the brain, which refers to the signal from motor areas that evoke self-generated movements [218]. Similarly, the decisions of mice under audiovisual conflicts, in which auditory and visual stimuli have opposite meanings, were measured in two different behavioural states: a head-restrained setup in a stationary state and a freely navigating setup in the T-maze [20,21]. Even though the perceptual discrimination performances were similar between the auditory and the visual stimuli, the mouse followed the auditory rules for the decisions in the head-restrained state while they followed the visual rules during navigation in the T-maze [20,21]. Therefore, corollary discharge in the brain may contribute to the different multisensory decisions depending on the locomotion state.

## 4. Changes in multisensory integration in neurodevelopmental disorders

Deficits in MSI are shown in a number of neurodevelopmental disorders, such as ASD [219], schizophrenia [220], developmental dyslexia [221] and attention-deficit/hyperactivity disorder (ADHD) [222]. In particular, ASD and schizophrenia are the most prevalent neurodevelopmental disorders accompanied by multiple behavioural abnormalities. Patients with ASD and schizophrenia show typical abnormalities in their behaviours. According to the Diagnostic and Statistical Manual of Mental Disorders-IV (DSM-IV) criteria [223], ASD patients show common symptoms such as persistent impairment in reciprocal social communication and interaction and restricted and repetitive behaviours [223,224]. On the other hand, schizophrenia patients show distinct symptoms such as delusions, hallucinations and disorganized speech [223,225]. Overall, both types of patients show deficits in sensory processing. ASD patients show hyper- or hyposensitivity to auditory, visual, tactile and olfactory stimuli, and these symptoms appear in various combinations across patients [226–230]. Patients with schizophrenia also often show hyper- or hyposensitivity to auditory and visual stimuli, and they sometimes experience distorted auditory and visual perception and hallucinations [231–235]. With such backgrounds, ASD and schizophrenia patients show similar or distinct impairments in sensory processing and perception (table 2). In this section, we describe abnormal MSI behaviours that appeared in these patients and neural mechanisms of such changes in MSI found in human patients and animal models with ASD and schizophrenia.

### (a) MSI deficits in autism

ASD patients not only have dysfunction in unisensory perception but they also have impairments in MSI. Many human studies have demonstrated abnormal audiovisual integration in ASD patients [73,77,81,87–91,219,236,237]. For instance, ASD children and teenagers (8–17 years old) who performed SIFI tasks showed stronger flash-beep illusions than normal children with typical development (TD) [81]. In the temporal order judgement task, during which the subject reports which one is presented first when visual and auditory stimuli were randomly given at intervals of 0 to hundreds of milliseconds, ASD children showed a wider temporal binding window and less sensitivity to timing differences [83,84]. Similarly, in the simultaneity judgement task (figure 1*b*), during which the subject determines whether the visual and the auditory stimuli were presented simultaneously or not, ASD children also showed a wider temporal binding window than TD children [77,78]. ASD patients also showed impairments in MSI during speech perception, and the McGurk effect was significantly reduced in ASD children compared to TD children [87–91,237].

Interestingly, unlike ASD children, older ASD patients (teenager to adult) showed a tendency to recover normal behaviours in these temporal or speech MSI tasks [219,236,238–240]. For example, multisensory speech recognition was impaired when ASD children (5–12 years old) were presented with random monosyllabic words spoken by a female speaker, but ASD teenagers (13–15 years old) showed normal performance in the same task [236]. In other types of MSI, however, such as the object integration task (figure 1*d*), ASD children and teenagers (8–19 years old) showed normal accuracies [87]. In another study using a causal inference model, ASD patients (15–17 years old) showed impaired causal inference of multisensory stimuli with a tendency to attribute auditory and visual stimuli from separate locations to a common source, although some compensatory mechanisms recovered their reports during an explicit task [241]. Therefore, not all MSI or MSI-related behaviours were disrupted in ASD, and this might be due to disruption in specific brain areas of MSI in ASD patients.

Although many human studies showed MSI changes only in their behavioural phenotypes in ASD patients, some studies have tried to find the underlying neural mechanisms of the MSI impairments [72,73,242]. For example, in a computational modelling study, researchers made a network model that explained the connection between the sensory cortex and the posterior STS, and they trained the network with the behavioural data from the McGurk experience of TD and ASD children [73]. They found that the decreased connectivity between the sensory cortex and the posterior STS cortex, due to fewer sensory inputs to the sensory cortex, explained the abnormal audiovisual integration behaviour in ASD patients [73]. Moreover, in a human EEG study, task-related *α* power (8–12 Hz), which increases when a subject suppresses the task-irrelevant sensory information with attention, was reduced in the parieto-occipital scalp of ASD patients [242]. Therefore, weakening in the connection between sensory cortices and the association cortex might cause impairments of MSI in ASD patients.

To reveal the neural mechanisms of ASD deficits in MSI, researchers examined several mouse models of ASD. For example, the serotonin transporter (SERT) mutant mice, known as an animal model of ASD, showed an impaired perceptual ability to detect audiovisual cues [72]. This might be due to impaired serotonergic secretion from the dorsal raphe nucleus to the SC [243], one of the core areas for multisensory processing [1]. Another example is the Cntnap2 knockout rats, known as ASD models, and they showed less sensitivity to temporal asynchrony in the temporal order judgement task [244]. However, the neural mechanism underlying this MSI change is still unclear. Furthermore, various mouse models of ASD, including BTBR T + tf/J, GAD65, Shank3 and Mecp2 knockout mice, developed abnormal E/I balance in the insular cortex, which showed reduced responses to the audio-tactile stimuli [74]. Future study is needed to determine if audiovisual integration is also altered in the insular cortex of these animals.

### (b) MSI deficits in schizophrenia

Schizophrenia patients also showed impairments in audiovisual integration [75,79,86,92,93,220,245,,246]. For example, during the speech perception test, schizophrenia patients rarely experienced the McGurk illusion [92–94]. They also experienced lower fusion illusions in the SIFI task [82]. Interestingly, schizophrenic patients showed normal MSI in spatial integration of audiovisual stimuli as tested by the ventriloquism effect test [86] but clear deficits in temporal MSI of audiovisual stimuli during the temporal order judgement and the simultaneity judgement tasks [79,80,85]. Therefore, schizophrenic patients show impairments in different types of MSI, which are particularly sensitive to the temporal aspects, compared with ASD patients (table 2). Some studies examined the ketamine-treated rodents as an animal model of schizophrenia and found MSI deficits in tactile–visual or olfactory–visual integrations [247,248]. Future research is needed to determine whether audiovisual integration is similarly disturbed in the animal model of schizophrenia.

Neural mechanisms of MSI deficits in schizophrenia are largely unknown. However, some human EEG and fMRI studies figured out the key brain areas that showed abnormal neural activities in schizophrenia patients when they performed audiovisual integration tasks [75,76,93,249]. These studies found that the fronto-temporal regions, including the IFG, the MTG and the STS [75], the medio-central scalp regions [93] and the occipital–parietal scalp regions [249] are potentially key regions that harbour MSI deficits in schizophrenia patients. Moreover, beta–gamma band power was reduced in the occipital region of schizophrenia patients during the SIFI test [76].

In summary, ASD and schizophrenia patients and animal models showed similar or distinct impairments in MSI processing. Both ASD and schizophrenia patients showed abnormal MSI and perception during the McGurk effect, SIFI, temporal order judgement and simultaneity judgement tasks (table 2). Behavioural phenotype in McGurk effect and simultaneity judgement tasks is similar between ASD and schizophrenia patients, but there are still abnormal but different results in SIFI and temporal order judgement tasks (table 2). In the object integration task and the spatial ventriloquism task, ASD and schizophrenia patients show normal behaviour, although not all tasks were tested (table 2). The common brain areas impaired in ASD and schizophrenia were the STS and STG [73,75]. Neural mechanisms of MSI changes in ASD and schizophrenia are still elusive, and further research is necessary to discover them.

## 5. Conclusion and future directions

In this review, we overviewed the multisensory areas in the mammalian brain (figure 1). We reviewed these areas based on their involvement in the five types of MSI measured in mammals, including humans, non-human primates and rodents (table 1). In awake and behaving animals, MSI can flexibly change depending on long- and short-term changes in the internal states of an animal as well as in the conditions of developing psychiatric disorders. Future studies are required to understand the neural circuit mechanism of each change in MSI and how the altered MSI in disease conditions has an impact on patients suffering from abnormal perception in complicated sensory environments.

## Data Availability

This article has no additional data.

## References

[1] Meredith MA, Stein BE. 1983 Interactions among converging sensory inputs in the superior colliculus. Science **221**, 389-391. (10.1126/science.6867718) 6867718

[2] Wallace MT, Stein BE. 1996 Sensory organization of the superior colliculus in cat and monkey. Prog. Brain Res. **112**, 301-311. (10.1016/S0079-6123(08)63337-3) 8979837

[3] Wallace MT, Wilkinson LK, Stein BE. 1996 Representation and integration of multiple sensory inputs in primate superior colliculus. J. Neurophysiol. **76**, 1246-1266. (10.1152/jn.1996.76.2.1246) 8871234

[4] Wallace MT, Meredith MA, Stein BE. 1998 Multisensory integration in the superior colliculus of the alert cat. J. Neurophysiol. **80**, 1006-1010. (10.1152/jn.1998.80.2.1006) 9705489

[5] Meredith MA, Stein BE. 1986 Visual, auditory, and somatosensory convergence on cells in superior colliculus results in multisensory integration. J. Neurophysiol. **56**, 640-662. (10.1152/jn.1986.56.3.640) 3537225

[6] Alvarado JC, Vaughan JW, Stanford TR, Stein BE. 2007 Multisensory versus unisensory integration: contrasting modes in the superior colliculus. J. Neurophysiol. **97**, 3193-3205. (10.1152/jn.00018.2007) 17329632

[7] Ghose D, Wallace MT. 2014 Heterogeneity in the spatial receptive field architecture of multisensory neurons of the superior colliculus and its effects on multisensory integration. Neuroscience **256**, 147-162. (10.1016/j.neuroscience.2013.10.044) 24183964 PMC3878431

[8] Meredith MA, Stein BE. 1996 Spatial determinants of multisensory integration in cat superior colliculus neurons. J. Neurophysiol. **75**, 1843-1857. (10.1152/jn.1996.75.5.1843) 8734584

[9] Ito S, Si Y, Litke AM, Feldheim DA. 2021 Nonlinear visuoauditory integration in the mouse superior colliculus. PLoS Comput. Biol. **17**, e1009181. (10.1371/journal.pcbi.1009181) 34723955 PMC8584769

[10] Alvarado JC, Stanford TR, Vaughan JW, Stein BE. 2007 Cortex mediates multisensory but not unisensory integration in superior colliculus. J. Neurosci. **27**, 12 775-12 786. (10.1523/JNEUROSCI.3524-07.2007) PMC6673293 18032649

[11] Jiang W, Stein BE. 2003 Cortex controls multisensory depression in superior colliculus. J. Neurophysiol. **90**, 2123-2135. (10.1152/jn.00369.2003) 14534263

[12] Jiang W, Wallace MT, Jiang H, Vaughan JW, Stein BE. 2001 Two cortical areas mediate multisensory integration in superior colliculus neurons. J. Neurophysiol. **85**, 506-522. (10.1152/jn.2001.85.2.506) 11160489

[13] Jiang W, Jiang H, Stein BE. 2006 Neonatal cortical ablation disrupts multisensory development in superior colliculus. J. Neurophysiol. **95**, 1380-1396. (10.1152/jn.00880.2005) 16267111 PMC1538963

[14] Wallace MT, Stein BE. 1997 Development of multisensory neurons and multisensory integration in cat superior colliculus. J. Neurosci. **17**, 2429-2444. (10.1523/JNEUROSCI.17-07-02429.1997) 9065504 PMC6573512

[15] Wallace MT, Stein BE. 2001 Sensory and multisensory responses in the newborn monkey superior colliculus. J. Neurosci. **21**, 8886-8894. (10.1523/JNEUROSCI.21-22-08886.2001) 11698600 PMC6762279

[16] Wallace MT, Perrault TJ, Hairston WD, Stein BE. 2004 Visual experience is necessary for the development of multisensory integration. J. Neurosci. **24**, 9580-9584. (10.1523/JNEUROSCI.2535-04.2004) 15509745 PMC6730167

[17] Yu L, Rowland BA, Stein BE. 2010 Initiating the development of multisensory integration by manipulating sensory experience. J. Neurosci. **30**, 4904-4913. (10.1523/JNEUROSCI.5575-09.2010) 20371810 PMC2858413

[18] Meredith MA, Nemitz JW, Stein BE. 1987 Determinants of multisensory integration in superior colliculus neurons. I. Temporal factors. J. Neurosci. **7**, 3215-3229. (10.1523/JNEUROSCI.07-10-03215.1987) 3668625 PMC6569162

[19] Molholm S, Sehatpour P, Mehta AD, Shpaner M, Gomez-Ramirez M, Ortigue S, Dyke JP, Schwartz TH, Foxe JJ. 2006 Audio-visual multisensory integration in superior parietal lobule revealed by human intracranial recordings. J. Neurophysiol. **96**, 721-729. (10.1152/jn.00285.2006) 16687619

[20] Song Y-H, Kim J-H, Jeong H-W, Choi I, Jeong D, Kim K, Lee S-H. 2017 A neural circuit for auditory dominance over visual perception. Neuron **93**, 940-954. (10.1016/j.neuron.2017.01.006) 28162806

[21] Choi I, Lee SH. 2022 Reduced activity of parvalbumin-positive interneurons in the posterior parietal cortex causes visually dominant multisensory decisions in freely navigating mice. Mol. Brain. **15**, 82. (10.1186/s13041-022-00968-x) 36224591 PMC9559816

[22] Barraclough NE, Xiao D, Baker CI, Oram MW, Perrett DI. 2005 Integration of visual and auditory information by superior temporal sulcus neurons responsive to the sight of actions. J. Cogn. Neurosci. **17**, 377-391. (10.1162/0898929053279586) 15813999

[23] Benevento LA, Fallon J, Davis BJ, Rezak M. 1977 Auditory–visual interaction in single cells in the cortex of the superior temporal sulcus and the orbital frontal cortex of the macaque monkey. Exp. Neurol. **57**, 849-872. (10.1016/0014-4886(77)90112-1) 411682

[24] Iurilli G, Ghezzi D, Olcese U, Lassi G, Nazzaro C, Tonini R, Tucci V, Benfenati F, Medini P. 2012 Sound-driven synaptic inhibition in primary visual cortex. Neuron **73**, 814-828. (10.1016/j.neuron.2011.12.026) 22365553 PMC3315003

[25] Meijer GT, Montijn JS, Pennartz CMA, Lansink CS. 2017 Audiovisual modulation in mouse primary visual cortex depends on cross-modal stimulus configuration and congruency. J. Neurosci. **37**, 8783-8796. (10.1523/JNEUROSCI.0468-17.2017) 28821672 PMC6596670

[26] Raij T et al. 2010 Onset timing of cross-sensory activations and multisensory interactions in auditory and visual sensory cortices. Eur. J. Neurosci. **31**, 1772-1782. (10.1111/j.1460-9568.2010.07213.x) 20584181 PMC3008317

[27] Mercier MR, Foxe JJ, Fiebelkorn IC, Butler JS, Schwartz TH, Molholm S. 2013 Auditory-driven phase reset in visual cortex: human electrocorticography reveals mechanisms of early multisensory integration. Neuroimage **79**, 19-29. (10.1016/j.neuroimage.2013.04.060) 23624493 PMC3677511

[28] Guerreiro MJ, Putzar L, Roder B. 2015 The effect of early visual deprivation on the neural bases of multisensory processing. Brain **138**, 1499-1504. (10.1093/brain/awv076) 25808371 PMC4614145

[29] Knöpfel T, Sweeney Y, Radulescu CI, Zabouri N, Doostdar N, Clopath C, Barnes SJ. 2019 Audio-visual experience strengthens multisensory assemblies in adult mouse visual cortex. Nat. Commun. **10**, 5684. (10.1038/s41467-019-13607-2) 31831751 PMC6908602

[30] Atilgan H, Town SM, Wood KC, Jones GP, Maddox RK, Lee AKC, Bizley JK. 2018 Integration of visual information in auditory cortex promotes auditory scene analysis through multisensory binding. Neuron **97**, 640-655. (10.1016/j.neuron.2017.12.034) 29395914 PMC5814679

[31] Kayser C, Logothetis NK, Panzeri S. 2010 Visual enhancement of the information representation in auditory cortex. Curr. Biol. **20**, 19-24. (10.1016/j.cub.2009.10.068) 20036538

[32] Kayser C, Petkov CI, Logothetis NK. 2008 Visual modulation of neurons in auditory cortex. Cereb. Cortex **18**, 1560-1574. (10.1093/cercor/bhm187) 18180245

[33] Kayser C, Petkov CI, Augath M, Logothetis NK. 2007 Functional imaging reveals visual modulation of specific fields in auditory cortex. J. Neurosci. **27**, 1824-1835. (10.1523/JNEUROSCI.4737-06.2007) 17314280 PMC6673538

[34] Noesselt T, Tyll S, Boehler CN, Budinger E, Heinze H-J, Driver J. 2010 Sound-induced enhancement of low-intensity vision: multisensory influences on human sensory-specific cortices and thalamic bodies relate to perceptual enhancement of visual detection sensitivity. J. Neurosci. **30**, 13 609-13 623. (10.1523/JNEUROSCI.4524-09.2010) 20943902 PMC2958511

[35] Komura Y, Tamura R, Uwano T, Nishijo H, Ono T. 2005 Auditory thalamus integrates visual inputs into behavioral gains. Nat. Neurosci. **8**, 1203-1209. (10.1038/nn1528) 16116444

[36] Bushara KO, Hanakawa T, Immisch I, Toma K, Kansaku K, Hallett M. 2003 Neural correlates of cross-modal binding. Nat. Neurosci. **6**, 190-195. (10.1038/nn993) 12496761

[37] van den Brink RL, Cohen MX, Van Der Burg E, Talsma D, Vissers ME, Slagter HA. 2013 Subcortical, modality-specific pathways contribute to multisensory processing in humans. Cereb. Cortex **24**, 2169-2177. (10.1093/cercor/bht069) 23529004

[38] Vittek A-L, Juan C, Nowak LG, Girard P, Cappe C. 2022 Multisensory integration in neurons of the medial pulvinar of macaque monkey. Cereb. Cortex **33**, 4202-4215. (10.1093/cercor/bhac337) PMC10110443 36068947

[39] Shan L, Yuan L, Zhang B, Ma J, Xu X, Gu F, Jiang Y, Dai J. 2023 Neural integration of audiovisual sensory inputs in macaque amygdala and adjacent regions. Neurosci. Bull. 1-13. (10.1007/s12264-022-00887-w) PMC10661144 36920645

[40] Dolan RJ, Morris JS, De Gelder B. 2001 Crossmodal binding of fear in voice and face. Proc. Natl Acad. Sci. USA **98**, 10 006-10 010. (10.1073/pnas.171288598) PMC55568 11493699

[41] Park JY, Gu BM, Kang DH, Shin YW, Choi CH, Lee JM, Kwon JS. 2010 Integration of cross-modal emotional information in the human brain: an fMRI study. Cortex **46**, 161-169. (10.1016/j.cortex.2008.06.008) 18691703

[42] Powers 3rd AR, Hevey MA, Wallace MT. 2012 Neural correlates of multisensory perceptual learning. J. Neurosci. **32**, 6263-6274. (10.1523/JNEUROSCI.6138-11.2012) 22553032 PMC3366559

[43] Naue N, Rach S, Strüber D, Huster RJ, Zaehle T, Körner U Herrmann CS. 2011 Auditory event-related response in visual cortex modulates subsequent visual responses in humans. J. Neurosci. **31**, 7729-7736. (10.1523/JNEUROSCI.1076-11.2011) 21613485 PMC6633117

[44] Kobayasi KI, Suwa Y, Riquimaroux H. 2013 Audiovisual integration in the primary auditory cortex of an awake rodent. Neurosci. Lett. **534**, 24-29. (10.1016/j.neulet.2012.10.056) 23142716

[45] Thorne JD, De Vos M, Viola FC, Debener S. 2011 Cross-modal phase reset predicts auditory task performance in humans. J. Neurosci. **31**, 3853-3861. (10.1523/JNEUROSCI.6176-10.2011) 21389240 PMC6622791

[46] Rohe T, Noppeney U. 2016 Distinct computational principles govern multisensory integration in primary sensory and association cortices. Curr. Biol. **26**, 509-514. (10.1016/j.cub.2015.12.056) 26853368

[47] Berger CC, Ehrsson HH. 2014 The fusion of mental imagery and sensation in the temporal association cortex. J. Neurosci. **34**, 13 684-13 692. (10.1523/JNEUROSCI.0943-14.2014) PMC4188966 25297095

[48] Callan A, Callan D, Ando H. 2015 An fMRI study of the ventriloquism effect. Cereb. Cortex **25**, 4248-4258. (10.1093/cercor/bhu306) 25577576 PMC4816779

[49] Bonath B, Noesselt T, Martinez A, Mishra J, Schwiecker K, Heinze H-J, Hillyard SA. 2007 Neural basis of the ventriloquist illusion. Curr. Biol. **17**, 1697-1703. (10.1016/j.cub.2007.08.050) 17884498

[50] Regenbogen C, Seubert J, Johansson E, Finkelmeyer A, Andersson P, Lundström JN. 2018 The intraparietal sulcus governs multisensory integration of audiovisual information based on task difficulty. Hum. Brain Mapp. **39**, 1313-1326. (10.1002/hbm.23918) 29235185 PMC6866436

[51] Werner S, Noppeney U. 2010 Distinct functional contributions of primary sensory and association areas to audiovisual integration in object categorization. J. Neurosci. **30**, 2662-2675. (10.1523/JNEUROSCI.5091-09.2010) 20164350 PMC6634553

[52] Diaconescu AO, Hasher L, Mcintosh AR. 2013 Visual dominance and multisensory integration changes with age. Neuroimage **65**, 152-166. (10.1016/j.neuroimage.2012.09.057) 23036447

[53] Beauchamp MS, Lee KE, Argall BD, Martin A. 2004 Integration of auditory and visual information about objects in superior temporal sulcus. Neuron **41**, 809-823. (10.1016/S0896-6273(04)00070-4) 15003179

[54] Stevenson RA, Geoghegan ML, James TW. 2007 Superadditive BOLD activation in superior temporal sulcus with threshold non-speech objects. Exp. Brain Res. **179**, 85-95. (10.1007/s00221-006-0770-6) 17109108

[55] Stevenson RA, James TW. 2009 Audiovisual integration in human superior temporal sulcus: inverse effectiveness and the neural processing of speech and object recognition. Neuroimage **44**, 1210-1223. (10.1016/j.neuroimage.2008.09.034) 18973818

[56] Naumer MJ, Doehrmann O, Muller NG, Muckli L, Kaiser J, Hein G. 2009 Cortical plasticity of audio-visual object representations. Cereb. Cortex **19**, 1641-1653. (10.1093/cercor/bhn200) 19015373 PMC2693620

[57] Thelen A, Cappe C, Murray MM. 2012 Electrical neuroimaging of memory discrimination based on single-trial multisensory learning. Neuroimage **62**, 1478-1488. (10.1016/j.neuroimage.2012.05.027) 22609795

[58] Thelen A, Talsma D, Murray MM. 2015 Single-trial multisensory memories affect later auditory and visual object discrimination. Cognition **138**, 148-160. (10.1016/j.cognition.2015.02.003) 25743256

[59] Laurienti PJ, Wallace MT, Maldjian JA, Susi CM, Stein BE, Burdette JH. 2003 Cross-modal sensory processing in the anterior cingulate and medial prefrontal cortices. Hum. Brain Mapp. **19**, 213-223. (10.1002/hbm.10112) 12874776 PMC6871917

[60] Diehl MM, Plakke BA, Albuquerque ER, Romanski LM. 2022 Representation of expression and identity by ventral prefrontal neurons. Neuroscience **496**, 243-260. (10.1016/j.neuroscience.2022.05.033) 35654293 PMC10363293

[61] Diaconescu AO, Alain C, Mcintosh AR. 2011 The co-occurrence of multisensory facilitation and cross-modal conflict in the human brain. J. Neurophysiol. **106**, 2896-2909. (10.1152/jn.00303.2011) 21880944

[62] Womelsdorf T, Anton-Erxleben K, Treue S. 2008 Receptive field shift and shrinkage in macaque middle temporal area through attentional gain modulation. J. Neurosci. **28**, 8934-8944. (10.1523/JNEUROSCI.4030-07.2008) 18768687 PMC6670861

[63] Calvert GA, Campbell R, Brammer MJ. 2000 Evidence from functional magnetic resonance imaging of crossmodal binding in the human heteromodal cortex. Curr. Biol. **10**, 649-657. (10.1016/S0960-9822(00)00513-3) 10837246

[64] Nath AR, Beauchamp MS. 2012 A neural basis for interindividual differences in the McGurk effect, a multisensory speech illusion. Neuroimage **59**, 781-787. (10.1016/j.neuroimage.2011.07.024) 21787869 PMC3196040

[65] Sekiyama K, Kanno I, Miura S, Sugita Y. 2003 Auditory-visual speech perception examined by fMRI and PET. Neurosci. Res. **47**, 277-287. (10.1016/S0168-0102(03)00214-1) 14568109

[66] Beauchamp MS, Nath AR, Pasalar S. 2010 fMRI-guided transcranial magnetic stimulation reveals that the superior temporal sulcus is a cortical locus of the McGurk effect. J. Neurosci. **30**, 2414-2417. (10.1523/JNEUROSCI.4865-09.2010) 20164324 PMC2844713

[67] Keil J, Muller N, Ihssen N, Weisz N. 2012 On the variability of the McGurk effect: audiovisual integration depends on prestimulus brain states. Cereb. Cortex **22**, 221-231. (10.1093/cercor/bhr125) 21625011

[68] Sugihara T, Diltz MD, Averbeck BB, Romanski LM. 2006 Integration of auditory and visual communication information in the primate ventrolateral prefrontal cortex. J. Neurosci. **26**, 11 138-11 147. (10.1523/JNEUROSCI.3550-06.2006) PMC2767253 17065454

[69] Homae F, Hashimoto R, Nakajima K, Miyashita Y, Sakai KL. 2002 From perception to sentence comprehension: the convergence of auditory and visual information of language in the left inferior frontal cortex. Neuroimage. **16**, 883-900. (10.1006/nimg.2002.1138) 12202077

[70] Ojanen V, Möttönen R, Pekkola J, Jääskeläinen IP, Joensuu R, Autti T, Sams M. 2005 Processing of audiovisual speech in Broca's area. Neuroimage **25**, 333-338. (10.1016/j.neuroimage.2004.12.001) 15784412

[71] Crosse MJ, Butler JS, Lalor EC. 2015 Congruent visual speech enhances cortical entrainment to continuous auditory speech in noise-free conditions. J. Neurosci. **35**, 14 195-14 204. (10.1523/JNEUROSCI.1829-15.2015) PMC6605423 26490860

[72] Siemann JK, Muller CL, Forsberg CG, Blakely RD, Veenstra-Vanderweele J, Wallace MT. 2017 An autism-associated serotonin transporter variant disrupts multisensory processing. Transl. Psychiatry. **7**, e1067. (10.1038/tp.2017.17) 28323282 PMC5416665

[73] Cuppini C, Ursino M, Magosso E, Ross LA, Foxe JJ, Molholm S. 2017 A computational analysis of neural mechanisms underlying the maturation of multisensory speech integration in neurotypical children and those on the autism spectrum. Front. Hum. Neurosci. **11**, 518. (10.3389/fnhum.2017.00518) 29163099 PMC5670153

[74] Gogolla N, Takesian AE, Feng G, Fagiolini M, Hensch TK. 2014 Sensory integration in mouse insular cortex reflects GABA circuit maturation. Neuron **83**, 894-905. (10.1016/j.neuron.2014.06.033) 25088363 PMC4177076

[75] Stekelenburg JJ, Maes JP, Van Gool AR, Sitskoorn M, Vroomen J. 2013 Deficient multisensory integration in schizophrenia: an event-related potential study. Schizophr. Res. **147**, 253-261. (10.1016/j.schres.2013.04.038) 23707640

[76] Balz J, Roa Romero Y, Keil J, Krebber M, Niedeggen M, Gallinat J, Senkowski D. 2016 Beta/Gamma oscillations and event-related potentials indicate aberrant multisensory processing in schizophrenia. Front. Psychol. **7**, 1896. (10.3389/fpsyg.2016.01896) 27999553 PMC5138197

[77] Stevenson RA, Siemann JK, Schneider BC, Eberly HE, Woynaroski TG, Camarata SM, Wallace MT. 2014 Multisensory temporal integration in autism spectrum disorders. J. Neurosci. **34**, 691-697. (10.1523/JNEUROSCI.3615-13.2014) 24431427 PMC3891950

[78] Noel JP, De Niear MA, Stevenson R, Alais D, Wallace MT. 2017 Atypical rapid audio-visual temporal recalibration in autism spectrum disorders. Autism Res. **10**, 121-129. (10.1002/aur.1633) 27156926 PMC10791168

[79] Stevenson RA, Park S, Cochran C, Mcintosh LG, Noel J-P, Barense MD, Ferber S, Wallace MT. 2017 The associations between multisensory temporal processing and symptoms of schizophrenia. Schizophr. Res. **179**, 97-103. (10.1016/j.schres.2016.09.035) 27746052 PMC5463449

[80] Foucher JR, Lacambre M, Pham BT, Giersch A, Elliott MA. 2007 Low time resolution in schizophrenia: Lengthened windows of simultaneity for visual, auditory and bimodal stimuli. Schizophr. Res. **97**, 118-127. (10.1016/j.schres.2007.08.013) 17884350

[81] Foss-Feig JH, Kwakye LD, Cascio CJ, Burnette CP, Kadivar H, Stone WL, Wallace MT. 2010 An extended multisensory temporal binding window in autism spectrum disorders. Exp. Brain Res. **203**, 381-389. (10.1007/s00221-010-2240-4) 20390256 PMC2871100

[82] Vanes LD, White TP, Wigton RL, Joyce D, Collier T, Shergill SS. 2016 Reduced susceptibility to the sound-induced flash fusion illusion in schizophrenia. Psychiatry Res. **245**, 58-65. (10.1016/j.psychres.2016.08.016) 27526318

[83] Kwakye LD, Foss-Feig JH, Cascio CJ, Stone WL, Wallace MT. 2011 Altered auditory and multisensory temporal processing in autism spectrum disorders. Front. Integr. Neurosci. **4**, 129. (10.3389/fnint.2010.00129) 21258617 PMC3024004

[84] De Boer-Schellekens L, Eussen M, Vroomen J. 2013 Diminished sensitivity of audiovisual temporal order in autism spectrum disorder. Front. Integr. Neurosci. **7**, 1-8. (10.3389/fnint.2013.00008) 23450453 PMC3583106

[85] De Boer-Schellekens L, Stekelenburg JJ, Maes JP, Van Gool AR, Vroomen J. 2014 Sound improves diminished visual temporal sensitivity in schizophrenia. Acta Psychol. **147**, 136-142. (10.1016/j.actpsy.2013.06.013) 23896561

[86] De Gelder B, Vroomen J, Annen L, Masthof E, Hodiamont P. 2003 Audio-visual integration in schizophrenia. Schizophr. Res. **59**, 211-218. (10.1016/S0920-9964(01)00344-9) 12414077

[87] Mongillo EA, Irwin JR, Whalen DH, Klaiman C, Carter AS, Schultz RT. 2008 Audiovisual processing in children with and without autism spectrum disorders. J. Autism Dev. Disord. **38**, 1349-1358. (10.1007/s10803-007-0521-y) 18307027

[88] Bebko JM, Schroeder JH, Weiss JA. 2014 The McGurk effect in children with autism and Asperger syndrome. Autism Res. **7**, 50-59. (10.1002/aur.1343) 24136870

[89] Iarocci G, Rombough A, Yager J, Weeks DJ, Chua R. 2010 Visual influences on speech perception in children with autism. Autism. **14**, 305-320. (10.1177/1362361309353615) 20591957

[90] Irwin JR, Tornatore LA, Brancazio L, Whalen DH. 2011 Can children with autism spectrum disorders ‘hear’ a speaking face? Child Dev. **82**, 1397-1403. (10.1111/j.1467-8624.2011.01619.x) 21790542 PMC3169706

[91] Woynaroski TG, Kwakye LD, Foss-Feig JH, Stevenson RA, Stone WL, Wallace MT. 2013 Multisensory speech perception in children with autism spectrum disorders. J. Autism Dev. Disord. **43**, 2891-2902. (10.1007/s10803-013-1836-5) 23624833 PMC3998667

[92] Grohn C, Norgren E, Eriksson L. 2022 A systematic review of the neural correlates of multisensory integration in schizophrenia. Schizophr Res. Cogn. **27**, 100219. (10.1016/j.scog.2021.100219) 34660211 PMC8502765

[93] Roa Romero Y, Keil J, Balz J, Niedeggen M, Gallinat J, Senkowski D. 2016 Alpha-band oscillations reflect altered multisensory processing of the McGurk illusion in schizophrenia. Front. Hum. Neurosci. **10**, 41. (10.3389/fnhum.2016.00041) 26903845 PMC4751891

[94] Pearl D, Yodashkin-Porat D, Katz N, Valevski A, Aizenberg D, Sigler M, Weizman A, Kikinzon L. 2009 Differences in audiovisual integration, as measured by McGurk phenomenon, among adult and adolescent patients with schizophrenia and age-matched healthy control groups. Compr. Psychiat. **50**, 186-192. (10.1016/j.comppsych.2008.06.004) 19216897

[95] Lewis JW, Van Essen DC. 2000 Corticocortical connections of visual, sensorimotor, and multimodal processing areas in the parietal lobe of the macaque monkey. J. Comp. Neurol. **428**, 112-137. (10.1002/1096-9861(20001204)428:1<112::AID-CNE8>3.0.CO;2-9) 11058227

[96] Romanski LM. 2012 Convergence of auditory, visual, and somatosensory information in ventral prefrontal cortex. In The neural bases of multisensory processes (eds MM Murray, MT Wallace), pp. 667-682. Boca Raton, FL: Frontiers in Neuroscience. 22593866

[97] Seltzer B, Pandya DN. 1994 Parietal, temporal, and occipita projections to cortex of the superior temporal sulcus in the rhesus monkey: a retrograde tracer study. J. Comparat. Neurol. **343**, 445-463. (10.1002/cne.903430308) 8027452

[98] Garner AR, Keller GB. 2022 A cortical circuit for audio-visual predictions. Nat. Neurosci. **25**, 98. (10.1038/s41593-021-00974-7) 34857950 PMC8737331

[99] Stein BE, Stanford TR, Rowland BA. 2014 Development of multisensory integration from the perspective of the individual neuron. Nat. Rev. Neurosci. **15**, 520-535. (10.1038/nrn3742) 25158358 PMC4215474

[100] Sparks DL. 1986 Translation of sensory signals into commands for control of saccadic eye movements: role of primate superior colliculus. Physiol. Rev. **66**, 118-171. (10.1152/physrev.1986.66.1.118) 3511480

[101] May PJ. 2006 The mammalian superior colliculus: laminar structure and connections. Prog. Brain Res. **151**, 321-378. (10.1016/S0079-6123(05)51011-2) 16221594

[102] Meredith MA, Ruth Clemo H. 1989 Auditory cortical projection from the anterior ectosylvian sulcus (Field AES) to the superior colliculus in the cat: an anatomical and electrophysiological study. J. Comparat. Neurol. **289**, 687-707. (10.1002/cne.902890412) 2592605

[103] Norita M, Mucke L, Benedek G, Albowitz B, Katoh Y, Creutzfeldt O. 1986 Connections of the anterior ectosylvian visual area (AEV). Exp. Brain Res. **62**, 225-240. (10.1007/BF00238842) 3709710

[104] Norita M, Kase M, Hoshino K, Meguro R, Funaki S, Hirano S, Mchaffie JG. 1996 Extrinsic and intrinsic connections of the cat's lateral suprasylvian visual area. Prog. Brain Res. **112**, 231-250. (10.1016/S0079-6123(08)63333-6) 8979833

[105] Norita M, Mchaffie JG, Shimizu H, Stein BE. 1991 The corticostriatal and corticotectal projections of the feline lateral suprasylvian cortex demonstrated with anterograde biocytin and retrograde fluorescent techniques. Neurosci. Res. **10**, 149-155. (10.1016/0168-0102(91)90037-Y) 1710043

[106] Jiang W, Jiang H, Stein BE. 2002 Two corticotectal areas facilitate multisensory orientation behavior. J. Cogn. Neurosci. **14**, 1240-1255. (10.1162/089892902760807230) 12495529

[107] Wilkinson LK, Meredith MA, Stein BE. 1996 The role of anterior ectosylvian cortex in cross-modality orientation and approach behavior. Exp. Brain Res. **112**, 1-10. (10.1007/BF00227172) 8951401

[108] Burnett LR, Stein BE, Chaponis D, Wallace MT. 2004 Superior colliculus lesions preferentially disrupt multisensory orientation. Neuroscience **124**, 535-547. (10.1016/j.neuroscience.2003.12.026) 14980725

[109] Brang D, Taich ZJ, Hillyard SA, Grabowecky M, Ramachandran VS. 2013 Parietal connectivity mediates multisensory facilitation. Neuroimage **78**, 396-401. (10.1016/j.neuroimage.2013.04.047) 23611862 PMC3672392

[110] Baumann O, Greenlee MW. 2006 Neural correlates of coherent audiovisual motion perception. Cereb. Cortex **17**, 1433-1443. (10.1093/cercor/bhl055) 16928890

[111] Lewis JW, Beauchamp MS, Deyoe EA. 2000 A comparison of visual and auditory motion processing in human cerebral cortex. Cereb. Cortex **10**, 873-888. (10.1093/cercor/10.9.873) 10982748

[112] Avillac M, Hamed SB, Duhamel JR. 2007 Multisensory integration in the ventral intraparietal area of the macaque monkey. J. Neurosci. **27**, 1922-1932. (10.1523/JNEUROSCI.2646-06.2007) 17314288 PMC6673547

[113] Schroeder CE, Foxe JJ. 2002 The timing and laminar profile of converging inputs to multisensory areas of the macaque neocortex. Cogn. Brain Res. **14**, 187-198. (10.1016/S0926-6410(02)00073-3) 12063142

[114] Schlack A, Sterbing-D'angelo SJ, Hartung K, Hoffmann KP, Bremmer F. 2005 Multisensory space representations in the macaque ventral intraparietal area. J. Neurosci. **25**, 4616-4625. (10.1523/JNEUROSCI.0455-05.2005) 15872109 PMC6725030

[115] Guipponi O, Wardak C, Ibarrola D, Comte J-C, Sappey-Marinier D, Pinã¨De S, Ben Hamed S. 2013 Multimodal convergence within the intraparietal sulcus of the macaque monkey. J. Neurosci. **33**, 4128-4139. (10.1523/JNEUROSCI.1421-12.2013) 23447621 PMC6619317

[116] Linden JF, Grunewald A, Andersen RA. 1999 Responses to auditory stimuli in macaque lateral intraparietal area II. Behavioral modulation. J. Neurophysiol. **82**, 343-358. (10.1152/jn.1999.82.1.343) 10400963

[117] Mazzoni P, Bracewell RM, Barash S, Andersen RA. 1996 Spatially tuned auditory responses in area LIP of macaques performing delayed memory saccades to acoustic targets. J. Neurophysiol. **75**, 1233-1241. (10.1152/jn.1996.75.3.1233) 8867131

[118] Oh SW et al. 2014 A mesoscale connectome of the mouse brain. Nature **508**, 207-214. (10.1038/nature13186) 24695228 PMC5102064

[119] Zingg B et al. 2014 Neural networks of the mouse neocortex. Cell **156**, 1096-1111. (10.1016/j.cell.2014.02.023) 24581503 PMC4169118

[120] Raposo D, Kaufman MT, Churchland AK. 2014 A category-free neural population supports evolving demands during decision-making. Nat. Neurosci. **17**, 1784-1792. (10.1038/nn.3865) 25383902 PMC4294797

[121] Oude Lohuis MN, Marchesi P, Pennartz CMA, Olcese U. 2022 Functional (ir)relevance of posterior parietal cortex during audiovisual change detection. J. Neurosci. **42**, 5229-5245. (10.1523/JNEUROSCI.2150-21.2022) 35641187 PMC9236290

[122] Beauchamp MS. 2005 See me, hear me, touch me: multisensory integration in lateral occipital-temporal cortex. Curr. Opin Neurobiol. **15**, 145-153. (10.1016/j.conb.2005.03.011) 15831395

[123] Petrides M, Pandya DN. 1988 Association fiber pathways to the frontal cortex from the superior temporal region in the rhesus monkey. J. Comparat. Neurol. **273**, 52-66. (10.1002/cne.902730106) 2463275

[124] Seltzer B, Pandya DN. 1989 Frontal lobe connections of the superior temporal sulcus in the rhesus monkey. J. Comparat. Neurol. **281**, 97-113. (10.1002/cne.902810108) 2925903

[125] Ó Scalaidhe SP, Wilson FAW, Goldman-Rakic PS. 1999 Face-selective neurons during passive viewing and working memory performance of rhesus monkeys: evidence for intrinsic specialization of neuronal coding. Cereb. Cortex **9**, 459-475. (10.1093/cercor/9.5.459) 10450891

[126] Romanski LM, Averbeck BB, Diltz M. 2005 Neural representation of vocalizations in the primate ventrolateral prefrontal cortex. J. Neurophysiol. **93**, 734-747. (10.1152/jn.00675.2004) 15371495

[127] Beer AL, Plank T, Greenlee MW. 2011 Diffusion tensor imaging shows white matter tracts between human auditory and visual cortex. Exp. Brain Res. **213**, 299-308. (10.1007/s00221-011-2715-y) 21573953

[128] Cappe C, Barone P. 2005 Heteromodal connections supporting multisensory integration at low levels of cortical processing in the monkey. Eur. J. Neurosci. **22**, 2886-2902. (10.1111/j.1460-9568.2005.04462.x) 16324124

[129] Clavagnier S, Falchier A, Kennedy H. 2004 Long-distance feedback projections to area V1: implications for multisensory integration, spatial awareness, and visual consciousness. Cogn. Affect. Behav. Neurosci. **4**, 117-126. (10.3758/CABN.4.2.117) 15460918

[130] Falchier A, Clavagnier S, Barone P, Kennedy H. 2002 Anatomical evidence of multimodal integration in primate striate cortex. J. Neurosci. **22**, 5749-5759. (10.1523/JNEUROSCI.22-13-05749.2002) 12097528 PMC6758216

[131] Falchier A, Schroeder CE, Hackett TA, Lakatos P, Nascimento-Silva S, Ulbert I, Karmos G, Smiley JF. 2010 Projection from visual areas V2 and prostriata to caudal auditory cortex in the monkey. Cereb. Cortex **20**, 1529-1538. (10.1093/cercor/bhp213) 19875677 PMC2882821

[132] Henschke JU, Noesselt T, Scheich H, Budinger E. 2015 Possible anatomical pathways for short-latency multisensory integration processes in primary sensory cortices. Brain Struct. Funct. **220**, 955-977. (10.1007/s00429-013-0694-4) 24384580

[133] Laramée ME, Kurotani T, Rockland KS, Bronchti G, Boire D. 2011 Indirect pathway between the primary auditory and visual cortices through layer V pyramidal neurons in V2L in mouse and the effects of bilateral enucleation. Eur. J. Neurosci. **34**, 65-78. (10.1111/j.1460-9568.2011.07732.x) 21676038

[134] Laramée ME, Rockland KS, Prince S, Bronchti G, Boire D. 2012 Principal component and cluster analysis of layer V pyramidal cells in visual and non-visual cortical areas projecting to the primary visual cortex of the mouse. Cereb. Cortex **23**, 714-728. (10.1093/cercor/bhs060) 22426333

[135] Rockland KS, Ojima H. 2003 Multisensory convergence in calcarine visual areas in macaque monkey. Int. J. Psychophysiol. **50**, 19-26. (10.1016/S0167-8760(03)00121-1) 14511833

[136] Barth DS, Goldberg N, Brett B, Di S. 1995 The spatiotemporal organization of auditory, visual, and auditory-visual evoked potentials in rat cortex. Brain Res. **678**, 177-190. (10.1016/0006-8993(95)00182-P) 7620886

[137] Benevento LA, Standage GP. 1983 The organization of projections of the retinorecipient and nonretinorecipient nuclei of the pretectal complex and layers of the superior colliculus to the lateral pulvinar and medial pulvinar in the macaque monkey. J. Comparat. Neurol. **217**, 307-336. (10.1002/cne.902170307) 6886056

[138] Gutierrez C, Cola MG, Seltzer B, Cusick C. 2000 Neurochemical and connectional organization of the dorsal pulvinar complex in monkeys. J. Comparat. Neurol. **419**, 61-86. (10.1002/(SICI)1096-9861(20000327)419:1<61::AID-CNE4>3.0.CO;2-I) 10717640

[139] Homman-Ludiye J, Mundinano IC, Kwan WC, Bourne JA. 2020 Extensive connectivity between the medial pulvinar and the cortex revealed in the marmoset monkey. Cereb. Cortex **30**, 1797-1812. (10.1093/cercor/bhz203) 31711181

[140] Romanski L, Giguere M, Bates J, Goldman-Rakic P. 1997 Topographic organization of medial pulvinar connections with the prefrontal cortex in the rhesus monkey. J. Comparat. Neurol. **379**, 313-332. (10.1002/(SICI)1096-9861(19970317)379:3<313::AID-CNE1>3.0.CO;2-6) 9067827

[141] Froesel M, Cappe C, Hamed SB. 2021 A multisensory perspective onto primate pulvinar functions. Neurosci. Biobehav. Rev. **125**, 231-243. (10.1016/j.neubiorev.2021.02.043) 33662442

[142] Yirmiya R, Hocherman S. 1987 Auditory- and movement-related neural activity interact in the pulvinar of the behaving rhesus monkey. Brain Res. **402**, 93-102. (10.1016/0006-8993(87)91051-1) 3828792

[143] Nguyen MN, Hori E, Matsumoto J, Tran AH, Ono T, Nishijo H. 2013 Neuronal responses to face-like stimuli in the monkey pulvinar. Eur. J. Neurosci. **37**, 35-51. (10.1111/ejn.12020) 23121157

[144] Barsy B et al. 2020 Associative and plastic thalamic signaling to the lateral amygdala controls fear behavior. Nat. Neurosci. **23**, 625-637. (10.1038/s41593-020-0620-z) 32284608

[145] Kang SJ et al. 2022 A central alarm system that gates multi-sensory innate threat cues to the amygdala. Cell Rep. **40**, 111222. (10.1016/j.celrep.2022.111222) 35977501 PMC9420642

[146] Salay LD, Ishiko N, Huberman AD. 2018 A midline thalamic circuit determines reactions to visual threat. Nature **557**, 183-189. (10.1038/s41586-018-0078-2) 29720647 PMC8442544

[147] Calder AJ, Lawrence AD, Young AW. 2001 Neuropsychology of fear and loathing. Nat. Rev. Neurosci. **2**, 352-363. (10.1038/35072584) 11331919

[148] Scott SK, Young AW, Calder AJ, Hellawell DJ, Aggleton JP, Johnsons M. 1997 Impaired auditory recognition of fear and anger following bilateral amygdala lesions. Nature **385**, 254-257. (10.1038/385254a0) 9000073

[149] Bach DR, Hurlemann R. 2015 Dolan RJ. Impaired threat prioritisation after selective bilateral amygdala lesions. Cortex **63**, 206-213. (10.1016/j.cortex.2014.08.017) 25282058 PMC4317193

[150] Sinnett S, Spence C, Soto-Faraco S. 2007 Visual dominance and attention: the Colavita effect revisited. Percept. Psychophys. **69**, 673-686. (10.3758/BF03193770) 17929691

[151] Spence C. 2009 Explaining the Colavita visual dominance effect. Prog. Brain Res. **176**, 245-258. (10.1016/S0079-6123(09)17615-X) 19733761

[152] Mallick DB, Magnotti JF, Beauchamp MS. 2015 Variability and stability in the McGurk effect: contributions of participants, stimuli, time, and response type. Psychon. Bull. Rev. **22**, 1299-1307. (10.3758/s13423-015-0817-4) 25802068 PMC4580505

[153] Magnotti JF, Dzeda KB, Wegner-Clemens K, Rennig J, Beauchamp MS. 2020 Weak observer-level correlation and strong stimulus-level correlation between the McGurk effect and audiovisual speech-in-noise: a causal inference explanation. Cortex **133**, 371-383. (10.1016/j.cortex.2020.10.002) 33221701 PMC8592674

[154] Feng G, Zhou B, Zhou W, Beauchamp MS, Magnotti JF. 2019 A laboratory study of the Mcgurk effect in 324 monozygotic and dizygotic twins. Front. Neurosci. **13**, 1029. (10.3389/fnins.2019.01029) 31636529 PMC6787151

[155] Murray MM, Lewkowicz DJ, Amedi A, Wallace MT. 2016 Multisensory processes: a balancing act across the lifespan. Trends Neurosci. **39**, 567-579. (10.1016/j.tins.2016.05.003) 27282408 PMC4967384

[156] Van Atteveldt N, Murray MM, Thut G, Schroeder CE. 2014 Multisensory integration: flexible use of general operations. Neuron **81**, 1240-1253. (10.1016/j.neuron.2014.02.044) 24656248 PMC4090761

[157] Hirst RJ, Stacey JE, Cragg L, Stacey PC, Allen HA. 2018 The threshold for the McGurk effect in audio-visual noise decreases with development. Sci. Rep. **8**, 12372. (10.1038/s41598-018-30798-8) 30120399 PMC6098036

[158] Wozny DR, Shams L. 2011 Computational characterization of visually induced auditory spatial adaptation. Front. Integr. Neurosci. **5**, 75. (10.3389/fnint.2011.00075) 22069383 PMC3208186

[159] Hong FF, Badde S, Landy MS. 2022 Repeated exposure to either consistently spatiotemporally congruent or consistently incongruent audiovisual stimuli modulates the audiovisual common-cause prior. Sci. Rep-Uk. **12**, 15532. (10.1038/s41598-022-19041-7) PMC9478143 36109544

[160] Bruns P, Maiworm M, Roder B. 2014 Reward expectation influences audiovisual spatial integration. Atten. Percept. Psychophys. **76**, 1815-1827. (10.3758/s13414-014-0699-y) 24874263

[161] Bean NL, Stein BE, Rowland BA. 2021 Stimulus value gates multisensory integration. Eur. J. Neurosci. **53**, 3142-3159. (10.1111/ejn.15167) 33667027 PMC8177070

[162] Powers 3rd AR, Hillock AR, Wallace MT. 2009 Perceptual training narrows the temporal window of multisensory binding. J. Neurosci. **29**, 12 265-12 274. (10.1523/JNEUROSCI.3501-09.2009) PMC2771316 19793985

[163] Setti A, Stapleton J, Leahy D, Walsh C, Kenny RA, Newell FN. 2014 Improving the efficiency of multisensory integration in older adults: audio-visual temporal discrimination training reduces susceptibility to the sound-induced flash illusion. Neuropsychologia **61**, 259-268. (10.1016/j.neuropsychologia.2014.06.027) 24983146

[164] Tang X, Wu J, Shen Y. 2016 The interactions of multisensory integration with endogenous and exogenous attention. Neurosci. Biobehav. Rev. **61**, 208-224. (10.1016/j.neubiorev.2015.11.002) 26546734 PMC4753360

[165] Talsma D, Senkowski D, Soto-Faraco S, Woldorff MG. 2010 The multifaceted interplay between attention and multisensory integration. Trends Cogn. Sci. **14**, 400-410. (10.1016/j.tics.2010.06.008) 20675182 PMC3306770

[166] De Meo R, Murray MM, Clarke S, Matusz PJ. 2015 Top-down control and early multisensory processes: chicken vs. egg. Front. Integr. Neurosci. **9**, 17. (10.3389/fnint.2015.00017) 25784863 PMC4347447

[167] Talsma D, Doty TJ, Woldorff MG. 2007 Selective attention and audiovisual integration: is attending to both modalities a prerequisite for early integration? Cereb. Cortex **17**, 679-690. (10.1093/cercor/bhk016) 16707740

[168] Zierul B, Tong J, Bruns P, Roder B. 2019 Reduced multisensory integration of self-initiated stimuli. Cognition **182**, 349-359. (10.1016/j.cognition.2018.10.019) 30389144

[169] Lewkowicz DJ. 2014 Early experience and multisensory perceptual narrowing. Dev. Psychobiol. **56**, 292-315. (10.1002/dev.21197) 24435505 PMC3953347

[170] Fagiolini M, Pizzorusso T, Berardi N, Domenici L, Maffei L. 1994 Functional postnatal development of the rat primary visual cortex and the role of visual experience: dark rearing and monocular deprivation. Vision Res. **34**, 709-720. (10.1016/0042-6989(94)90210-0) 8160387

[171] Zhang LI, Bao S, Merzenich MM. 2001 Persistent and specific influences of early acoustic environments on primary auditory cortex. Nat. Neurosci. **4**, 1123-1130. (10.1038/nn745) 11687817

[172] Carriere BN, Royal DW, Perrault TJ, Morrison SP, Vaughan JW, Stein BE, Wallace MT. 2007 Visual deprivation alters the development of cortical multisensory integration. J. Neurophysiol. **98**, 2858-2867. (10.1152/jn.00587.2007) 17728386

[173] Putzar L, Goerendt I, Lange K, Rosler F, Roder B. 2007 Early visual deprivation impairs multisensory interactions in humans. Nat. Neurosci. **10**, 1243-1245. (10.1038/nn1978) 17873871

[174] Lewkowicz DJ, Leo I, Simion F. 2010 Intersensory perception at birth: newborns match nonhuman primate faces and voices. Infancy **15**, 46-60. (10.1111/j.1532-7078.2009.00005.x) 32693457

[175] Lewkowicz DJ, Turkewitz G. 1980 Cross-modal equivalence in early infancy: auditory–visual intensity matching. Dev. Psychol. **16**, 597-607. (10.1037/0012-1649.16.6.597)

[176] Bahrick LE, Lickliter R. 2000 Intersensory redundancy guides attentional selectivity and perceptual learning in infancy. Dev. Psychol. **36**, 190-201. (10.1037/0012-1649.36.2.190) 10749076 PMC2704001

[177] Lewkowicz DJ. 1996 Perception of auditory–visual temporal synchrony in human infants. J. Exp. Psychol. Hum. Percept. Perform. **22**, 1094-1106. (10.1037/0096-1523.22.5.1094) 8865617

[178] Scheier C, Lewkowicz DJ, Shimojo S. 2003 Sound induces perceptual reorganization of an ambiguous motion display in human infants. Dev. Sci. **6**, 233-241. (10.1111/1467-7687.00276)

[179] Lewkowicz DJ, Ghazanfar AA. 2009 The emergence of multisensory systems through perceptual narrowing. Trends Cogn. Sci. **13**, 470-478. (10.1016/j.tics.2009.08.004) 19748305

[180] Walker-Andrews AS. 1997 Infants' perception of expressive behaviors: differentiation of multimodal information. Psychol. Bull. **121**, 437-456. (10.1037/0033-2909.121.3.437) 9136644

[181] Lewkowicz DJ, Hansen-Tift AM. 2012 Infants deploy selective attention to the mouth of a talking face when learning speech. Proc. Natl Acad. Sci. USA **109**, 1431-1436. (10.1073/pnas.1114783109) 22307596 PMC3277111

[182] Rosenblum LD, Schmuckler MA, Johnson JA. 1997 The McGurk effect in infants. Percept. Psychophys. **59**, 347-357. (10.3758/BF03211902) 9136265

[183] Lewkowicz DJ, Pons F. 2013 Recognition of amodal language identity emerges in infancy. Int. J. Behav. Dev. **37**, 90-94. (10.1177/0165025412467582) 24648601 PMC3956126

[184] Lewkowicz DJ, Minar NJ, Tift AH, Brandon M. 2015 Perception of the multisensory coherence of fluent audiovisual speech in infancy: its emergence and the role of experience. J. Exp. Child Psychol. **130**, 147-162. (10.1016/j.jecp.2014.10.006) 25462038 PMC4258456

[185] Robinson CW, Sloutsky VM. 2004 Auditory dominance and its change in the course of development. Child Dev. **75**, 1387-1401. (10.1111/j.1467-8624.2004.00747.x) 15369521

[186] Hirst RJ, Cragg L, Allen HA. 2018 Vision dominates audition in adults but not children: a meta-analysis of the Colavita effect. Neurosci. Biobehav. Rev. **94**, 286-301. (10.1016/j.neubiorev.2018.07.012) 30048672

[187] Brandwein AB, Foxe JJ, Russo NN, Altschuler TS, Gomes H, Molholm S. 2011 The development of audiovisual multisensory integration across childhood and early adolescence: a high-density electrical mapping study. Cereb. Cortex **21**, 1042-1055. (10.1093/cercor/bhq170) 20847153 PMC3077428

[188] Laurienti PJ, Burdette JH, Maldjian JA, Wallace MT. 2006 Enhanced multisensory integration in older adults. Neurobiol. Aging. **27**, 1155-1163. (10.1016/j.neurobiolaging.2005.05.024) 16039016

[189] Park H, Nannt J, Kayser C. 2021 Sensory- and memory-related drivers for altered ventriloquism effects and aftereffects in older adults. Cortex **135**, 298-310. (10.1016/j.cortex.2020.12.001) 33422888 PMC7856550

[190] Knill DC, Pouget A. 2004 The Bayesian brain: the role of uncertainty in neural coding and computation. Trends Neurosci. **27**, 712-719. (10.1016/j.tins.2004.10.007) 15541511

[191] Fetsch CR, Deangelis GC, Angelaki DE. 2013 Bridging the gap between theories of sensory cue integration and the physiology of multisensory neurons. Nat. Rev. Neurosci. **14**, 429-442. (10.1038/nrn3503) 23686172 PMC3820118

[192] Deneve S, Pouget A. 2004 Bayesian multisensory integration and cross-modal spatial links. J. Physiol. Paris. **98**, 249-258. (10.1016/j.jphysparis.2004.03.011) 15477036

[193] Magnotti JF, Beauchamp MS. 2017 A causal inference model explains perception of the McGurk effect and other incongruent audiovisual speech. PLoS Comput. Biol. **13**, e1005229. (10.1371/journal.pcbi.1005229) 28207734 PMC5312805

[194] Alais D, Burr D. 2004 The ventriloquist effect results from near-optimal bimodal integration. Curr. Biol. **14**, 257-262. (10.1016/j.cub.2004.01.029) 14761661

[195] Meijer D, Veselic S, Calafiore C, Noppeney U. 2019 Integration of audiovisual spatial signals is not consistent with maximum likelihood estimation. Cortex **119**, 74-88. (10.1016/j.cortex.2019.03.026) 31082680 PMC6864592

[196] Recanzone GH. 1998 Rapidly induced auditory plasticity: the ventriloquism aftereffect. Proc. Natl Acad. Sci. USA **95**, 869-875. (10.1073/pnas.95.3.869) 9448253 PMC33810

[197] Hertz U, Amedi A. 2015 Flexibility and stability in sensory processing revealed using visual-to-auditory sensory substitution. Cereb. Cortex **25**, 2049-2064. (10.1093/cercor/bhu010) 24518756 PMC4494022

[198] Lehmann S, Murray MM. 2005 The role of multisensory memories in unisensory object discrimination. Cogn. Brain Res. **24**, 326-334. (10.1016/j.cogbrainres.2005.02.005) 15993770

[199] Chang S, Xu J, Zheng M, Keniston L, Zhou X, Zhang J, Yu L. 2022 Integrating visual information into the auditory cortex promotes sound discrimination through choice-related multisensory integration. J. Neurosci. **42**, 8556-8568. (10.1523/JNEUROSCI.0793-22.2022) 36150889 PMC9665927

[200] Han X, Xu JH, Chang S, Keniston L, Yu LP. 2022 Multisensory-guided associative learning enhances multisensory representation in primary auditory cortex. Cereb. Cortex **32**, 1040-1054. (10.1093/cercor/bhab264) 34378017

[201] Binder JR, Desai RH, Graves WW, Conant LL. 2009 Where is the semantic system? A critical review and meta-analysis of 120 functional neuroimaging studies. Cereb. Cortex **19**, 2767-2796. (10.1093/cercor/bhp055) 19329570 PMC2774390

[202] Pashler HE. 1998. The psychology of attention. Cambridge, MA: MIT Press.

[203] Van Der Burg E, Olivers CN, Bronkhorst AW, Theeuwes J. 2008 Pip and pop: nonspatial auditory signals improve spatial visual search. J. Exp. Psychol. Hum. Percept. Perform. **34**, 1053-1065. (10.1037/0096-1523.34.5.1053) 18823194

[204] Theeuwes J. 1991 Exogenous and endogenous control of attention: the effect of visual onsets and offsets. Percept. Psychophys. **49**, 83-90. (10.3758/BF03211619) 2011456

[205] Calvert G, Spence C, Stein BE. 2004. In The handbook of multisensory processes. Cambridge, MA: MIT Press.

[206] Spence C. 2013 Just how important is spatial coincidence to multisensory integration? Evaluating the spatial rule. Ann. NY Acad. Sci. **1296**, 31-49. (10.1111/nyas.12121) 23710729

[207] Fritz JB, Elhilali M, David SV, Shamma SA. 2007 Does attention play a role in dynamic receptive field adaptation to changing acoustic salience in Al? Hearing Res. **229**, 186-203. (10.1016/j.heares.2007.01.009) PMC2077083 17329048

[208] Anton-Erxleben K, Stephan VM, Treue S. 2009 Attention reshapes center-surround receptive field structure in macaque cortical area MT. Cereb. Cortex **19**, 2466-2478. (10.1093/cercor/bhp002) 19211660 PMC2742598

[209] Stein BE, Stanford TR. 2008 Multisensory integration: current issues from the perspective of the single neuron. Nat. Rev. Neurosci. **9**, 255-266. (10.1038/nrn2331) 18354398

[210] Fairhall SL, Macaluso E. 2009 Spatial attention can modulate audiovisual integration at multiple cortical and subcortical sites. Eur. J. Neurosci. **29**, 1247-1257. (10.1111/j.1460-9568.2009.06688.x) 19302160

[211] Hugenschmidt CE, Mozolic JL, Laurienti PJ. 2009 Suppression of multisensory integration by modality-specific attention in aging. Neuroreport **20**, 349-353. (10.1097/WNR.0b013e328323ab07) 19218871 PMC2692738

[212] Mozolic JL, Hugenschmidt CE, Peiffer AM, Laurienti PJ. 2008 Modality-specific selective attention attenuates multisensory integration. Exp. Brain Res. **184**, 39-52. (10.1007/s00221-007-1080-3) 17684735

[213] Michail G, Senkowski D, Niedeggen M, Keil J. 2021 Memory load alters perception-related neural oscillations during multisensory integration. J. Neurosci. **41**, 1505-1515. (10.1523/JNEUROSCI.1397-20.2020) 33310755 PMC7896008

[214] Barutchu A, Spence C. 2021 Top–down task-specific determinants of multisensory motor reaction time enhancements and sensory switch costs. Exp. Brain Res. **239**, 1021-1034. (10.1007/s00221-020-06014-3) 33515085 PMC7943519

[215] Repp BH, Penel A. 2002 Auditory dominance in temporal processing: new evidence from synchronization with simultaneous visual and auditory sequences. J. Exp. Psychol. Hum. Percept. Perform. **28**, 1085-1099. (10.1037/0096-1523.28.5.1085) 12421057

[216] Vroomen J, Bertelson P, De Gelder B. 2001 The ventriloquist effect does not depend on the direction of automatic visual attention. Percept. Psychophys. **63**, 651-659. (10.3758/BF03194427) 11436735

[217] Welch RB, Warren DH. 1980 Immediate perceptual response to intersensory discrepancy. Psychol. Bull. **88**, 638-667. (10.1037/0033-2909.88.3.638) 7003641

[218] Crapse TB, Sommer MA. 2008 Corollary discharge across the animal kingdom. Nat. Rev. Neurosci. **9**, 587-600. (10.1038/nrn2457) 18641666 PMC5153363

[219] Beker S, Foxe JJ, Molholm S. 2018 Ripe for solution: delayed development of multisensory processing in autism and its remediation. Neurosci. Biobehav. Rev. **84**, 182-192. (10.1016/j.neubiorev.2017.11.008) 29162518 PMC6389331

[220] Ross LA, Saint-Amour D, Leavitt VM, Molholm S, Javitt DC, Foxe JJ. 2007 Impaired multisensory processing in schizophrenia: deficits in the visual enhancement of speech comprehension under noisy environmental conditions. Schizophr. Res. **97**, 173-183. (10.1016/j.schres.2007.08.008) 17928202

[221] Francisco AA, Jesse A, Groen MA, Mcqueen JM. 2017 A general audiovisual temporal processing deficit in adult readers with dyslexia. J. Speech Lang. Hear Res. **60**, 144-158. (10.1044/2016_JSLHR-H-15-0375) 28056152

[222] Reynolds S, Lane SJ. 2009 Sensory overresponsivity and anxiety in children with ADHD. Am. J. Occup. Ther. **63**, 433-440. (10.5014/ajot.63.4.433) 19708472

[223] Cooper J. 2001 Diagnostic and statistical manual of mental disorders (4th edn, text revision) (DSM-IV-TR). Washington, DC: American Psychiatric Association. (10.1192/bjp.179.1.85-a)

[224] Sharma SR, Gonda X, Tarazi FI. 2018 Autism spectrum disorder: classification, diagnosis and therapy. Pharmacol. Ther. **190**, 91-104. (10.1016/j.pharmthera.2018.05.007) 29763648

[225] Patel KR, Cherian J, Gohil K, Atkinson D. 2014 Schizophrenia: overview and treatment options. Pharmacy Ther. **39**, 638-645. PMC4159061 25210417

[226] Kanner L. 1968 Autistic disturbances of affective contact. Acta Paedopsychiatr. **35**, 100-136. 4880460

[227] Kern JK, Trivedi MH, Garver CR, Grannemann BD, Andrews AA, Savla JS, Johnson DG, Mehta JA, Schroeder JL. 2006 The pattern of sensory processing abnormalities in autism. Autism **10**, 480-494. (10.1177/1362361306066564) 16940314

[228] Leekam SR, Nieto C, Libby SJ, Wing L, Gould J. 2007 Describing the sensory abnormalities of children and adults with autism. J. Autism Dev. Disord. **37**, 894-910. (10.1007/s10803-006-0218-7) 17016677

[229] Marco EJ, Hinkley LB, Hill SS, Nagarajan SS. 2011 Sensory processing in autism: a review of neurophysiologic findings. Pediatr. Res. **69**, 48-54. (10.1203/PDR.0b013e3182130c54) PMC3086654 21289533

[230] Sinclair D, Oranje B, Razak KA, Siegel SJ, Schmid S. 2017 Sensory processing in autism spectrum disorders and Fragile X syndrome—from the clinic to animal models. Neurosci. Biobehav. Rev. **76**, 235-253. (10.1016/j.neubiorev.2016.05.029) 27235081 PMC5465967

[231] Javitt DC. 2009 When doors of perception close: bottom-up models of disrupted cognition in Schizophrenia. Annu. Rev. Clin. Psycho. **5**, 249-275. (10.1146/annurev.clinpsy.032408.153502) PMC4501390 19327031

[232] Javitt DC, Freedman R. 2015 Sensory processing dysfunction in the personal experience and neuronal machinery of schizophrenia. Am. J. Psychiatry **172**, 17-31. (10.1176/appi.ajp.2014.13121691) 25553496 PMC4501403

[233] Klosterkotter J, Schultze-Lutter F, Ruhrmann S. 2008 Kraepelin and psychotic prodromal conditions. Eur. Arch. Psychiatry Clin. Neurosci. **258**, 74-84. (10.1007/s00406-008-2010-5) 18516519

[234] Kompus K, Westerhausen R, Hugdahl K. 2011 The ‘paradoxical’ engagement of the primary auditory cortex in patients with auditory verbal hallucinations: a meta-analysis of functional neuroimaging studies. Neuropsychologia **49**, 3361-3369. (10.1016/j.neuropsychologia.2011.08.010) 21872614

[235] Mckay CM, Headlam DM, Copolov DL. 2000 Central auditory processing in patients with auditory hallucinations. Am. J. Psychiatry **157**, 759-766. (10.1176/appi.ajp.157.5.759) 10784469

[236] Foxe JJ, Molholm S, Del Bene VA, Frey H-P, Russo NN, Blanco D, Saint-Amour D, Ross LA. 2015 Severe multisensory speech integration deficits in high-functioning school-aged children with autism spectrum disorder (ASD) and their resolution during early adolescence. Cereb. Cortex **25**, 298-312. (10.1093/cercor/bht213) 23985136 PMC4303800

[237] Williams JH, Massaro DW, Peel NJ, Bosseler A, Suddendorf T. 2004 Visual–auditory integration during speech imitation in autism. Res. Dev. Disabil. **25**, 559-575. (10.1016/j.ridd.2004.01.008) 15541632

[238] Keane BP, Rosenthal O, Chun NH, Shams L. 2010 Audiovisual integration in high functioning adults with autism. Res. Autism Spect. Dis. **4**, 276-289. (10.1016/j.rasd.2009.09.015)

[239] Poole D, Gowen E, Warren PA, Poliakoff E. 2017 Brief Report: Which came first? Exploring crossmodal temporal order judgements and their relationship with sensory reactivity in autism and neurotypicals. J. Autism Dev. Disord. **47**, 215-223. (10.1007/s10803-016-2925-z) 27704294 PMC5222899

[240] Van Der Smagt MJ, Van Engeland H, Kemner C. 2007 Brief report: Can you see what is not there? Low-level auditory–visual integration in autism spectrum disorder. J. Autism Dev. Disord. **37**, 2014-2019. (10.1007/s10803-006-0346-0) 17273934

[241] Noel JP, Shivkumar S, Dokka K, Haefner RM, Angelaki DE. 2022 Aberrant causal inference and presence of a compensatory mechanism in autism spectrum disorder. Elife **11**, e71866. (10.7554/eLife.71866) 35579424 PMC9170250

[242] Murphy JW, Foxe JJ, Peters JB, Molholm S. 2014 Susceptibility to distraction in autism spectrum disorder: probing the integrity of oscillatory alpha-band suppression mechanisms. Autism Res. **7**, 442-458. (10.1002/aur.1374) 24678054 PMC4183200

[243] Anden NE, Dahlstrom A, Fuxe K, Larsson K. 1965 Mapping out of catecholamine and 5-hydroxytryptamine neurons innervating the telencephalon and diencephalon. Life Sci. **4**, 1275-1279. (10.1016/0024-3205(65)90076-7) 5849269

[244] Scott KE, Kazazian K, Mann RS, Möhrle D, Schormans AL, Schmid S, Allman BL. 2020 Loss of *Cntnap2* in the rat causes autism-related alterations in social interactions, stereotypic behavior, and sensory processing. Autism Res. **13**, 1698-1717. (10.1002/aur.2364) 32918359

[245] De Jong JJ, Hodiamont PP, Van Den Stock J, De Gelder B. 2009 Audiovisual emotion recognition in schizophrenia: reduced integration of facial and vocal affect. Schizophr. Res. **107**, 286-293. (10.1016/j.schres.2008.10.001) 18986799

[246] Wynn JK, Jahshan C, Green MF. 2014 Multisensory integration in schizophrenia: a behavioural and event-related potential study. Cogn. Neuropsychiatry **19**, 319-336. (10.1080/13546805.2013.866892) 24397788 PMC4103881

[247] Cloke JM, Nguyen R, Chung BYT, Wasserman DI, De Lisio S, Kim JC, Bailey CDC, Winters BD. 2016 A novel multisensory integration task reveals robust deficits in rodent models of schizophrenia: converging evidence for remediation via nicotinic receptor stimulation of inhibitory transmission in the prefrontal cortex. J. Neurosci. **36**, 12 570-12 585. (10.1523/JNEUROSCI.1628-16.2016) PMC6705662 27974613

[248] Jacklin DL, Goel A, Clementino KJ, Hall AW, Talpos JC, Winters BD. 2012 Severe cross-modal object recognition deficits in rats treated sub-chronically with NMDA receptor antagonists are reversed by systemic nicotine: implications for abnormal multisensory integration in schizophrenia. Neuropsychopharmacology **37**, 2322-2331. (10.1038/npp.2012.84) 22669170 PMC3422496

[249] Liu T, Pinheiro AP, Zhao Z, Nestor PG, Mccarley RW, Niznikiewicz M. 2016 Simultaneous face and voice processing in schizophrenia. Behav. Brain Res. **305**, 76-86. (10.1016/j.bbr.2016.01.039) 26804362

